# Dipyridinophane ligands – synthesis and coordination study

**DOI:** 10.1039/d6ra00469e

**Published:** 2026-04-07

**Authors:** Lucie Kuncová, Jana Lazarová, Jan Kotek, Vojtěch Kubíček, Petr Hermann

**Affiliations:** a Department of Inorganic Chemistry, Faculty of Science, Charles University in Prague Hlavova 2030 128 40 Prague Czech Republic kubicek@natur.cuni.cz

## Abstract

Twelve-membered dipyridinophane and its derivatives bearing two acetate or methylphosphonate pendant arms were synthesised, and their coordination properties were studied. Protonation constants of the ligands and stability constants of their complexes with Ni^II^, Cu^II^ and Zn^II^ ions were determined through a combination of potentiometry and spectral measurements. The ligands demonstrate significantly lower basicity than other tetraazamacrocycles (cyclen, pyclen) due to the presence of two pyridine units. This low macrocycle basicity results in high conditional stabilities of the complexes in acidic solutions. The solid-state structures of the complexes reveal that divalent first-row transition metal ions form a distorted octahedron with weakly coordinated pendant arms and bent coordination of the pyridine rings, as these metal ions are too large for the ligand cavity. Conversely, the ligand cavity is very suitable for smaller trivalent metal ions, and the complexes adopt an octahedral (Co^III^) or pentagonal bipyramidal (Fe^III^ and Ga^III^) arrangements.

## Abstract 2

Dipyridinophane derivatives show high coordination ability in acidic solutions. The geometry of the complexes is significantly influenced by the size of the metal ion.

## Graphical abstract

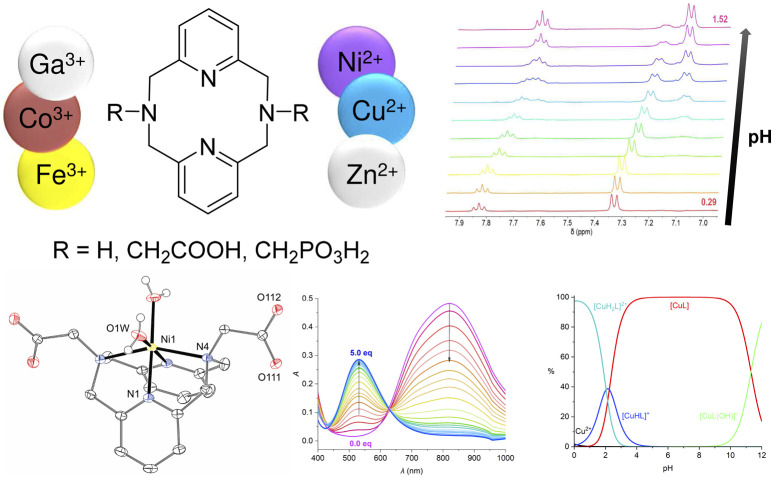

## Introduction

Azamacrocycles are an important class of ligands widely utilised for the complexation of metal ions. Many azamacrocyclic complexes are studied and utilised as contrast and therapeutic agents in medicine (magnetic resonance imaging, radiomedicine, *etc.*) and as catalysts or sensors in industry.^1–3^ A choice of suitable macrocyclic ligands leads to the complexes endowed with a high stability and a high inertness, as the macrocyclic complexes are often resistant towards acid- or base-catalysed dissociation, transmetallation or transchelation. The high inertness, which is the most important benefit of the macrocyclic complexes, is often crucial for medicinal applications as inert complexes remain intact or undergo a very slow dissociation, even under highly challenging conditions.^4,5^

**Fig. 1:**
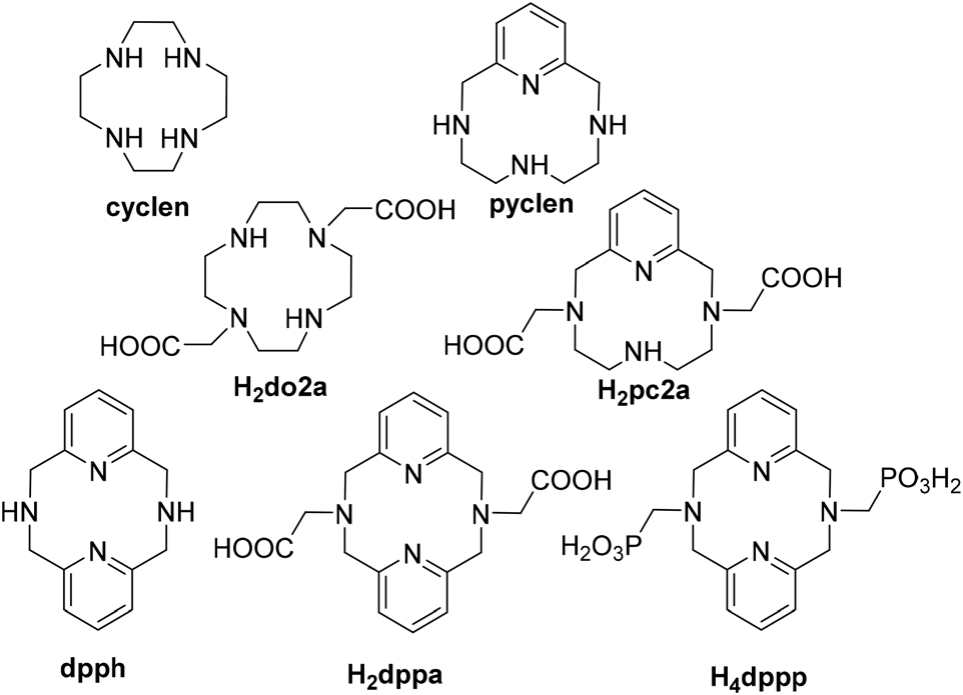
Ligands mentioned in the text.

The inertness can be improved by introducing rigid fragments into the macrocyclic scaffold. Thus, pyridine-containing macrocycles have attracted increasing attention in recent years.^6^ The derivatives of the most extensively studied pyridine-containing macrocycle, pyclen (Fig. 1), have been studied as metal carriers in magnetic resonance imaging, radiomedicine, or optical imaging.^7^ The analogous macrocycle with two pyridine units, dipyridinophane (dpph, Fig. 1), attracted less attention.^7,8^ The parent macrocycle dpph and, mainly, its *N*,*N*′-dialkylated derivatives were synthesized and their complexes were studied as catalysts of coupling reactions,^9^ catechol oxidation or DNA cleavage,^10,11^ CO_2_ reduction,^12^ water oxidation,^13,14^ and H_2_O_2_ disproportionation.^15–18^

Complexes of dpph show various coordination modes. In complexes of 1 : 1 metal-to-ligand stoichiometry, the ligand molecule is mostly coordinated in the tetradentate fashion. It was found in the manganese(ii)/(iii)/(iv),^16–18^ iron(iii),^10,12^ cobalt(iii),^12^ nickel(ii),^12,19^ copper(i)/(ii),^20–23^ zinc(ii),^12^ ruthenium(ii),^24^ or uranyl(vi)^25^ complexes. However, tridentate ligand coordination was also observed in the Cu^I^ complex.^23^ Several complexes with two ligand molecules coordinated to the metal ion were also structurally characterised. The metal ion bound to two tetracoordinated ligands (*i.e.*, coordination number 8) was found in the Fe^II^, Fe^III^ and Co^II^ complexes, whereas the Ni^II^ complex was hexacoordinated with one tetracoordinated and one dicoordinated ligand.^26,27^ The tetradentate coordination is also mostly preserved in a number of complexes with *N*,*N*′-dialkylated derivatives.^28^

Macrocycles are often modified with coordinating pendant arms in order to improve complexing ability and/or properties of the complexes. The dicarboxylate derivative H_2_dppa (Fig. 1) was synthesised, and its protonation constants and stability constants with Mg^II^, Ca^II^ and Gd^III^ ions were determined.^29^ Later, H_2_dppa labelling with ^64^Cu was studied and compared with the dipyridinophane derivatives bearing modified phenolate pendant arms for targeting amyloid plaques associated with Alzheimer's disease.^30,31^ Cobalt and copper complexes with the derivatives bearing pendant methylpyridyl groups were studied as catalysts for H_2_ evolution,^32^ or as superoxide dismutase mimics.^20^ The derivatives bearing one or two picolinate pendant arms were studied as carriers of ^52^Mn for PET,^33^ and for a complexation of the Gd^III^ ion in MRI.^34,35^

Despite various complexes of dpph and its derivatives were studied as metal ion carriers in the above-mentioned applications, there is still a lack of information on the coordination behaviour of this ligand class, mainly those bearing coordinating pendant arms. To fill this gap, we present here the results of a systematic study on acid–base and coordination behaviour of the parent macrocycle (dpph), its diacetic acid (H_2_dppa) and bis(methylphosphonic acid) (H_4_dppp) derivatives (Fig. 1). The coordination study was designed with respect to the potential application in biomedicine and was focused mainly at the transition metals present in the living organisms (Cu, Co, Ni, Zn, Fe) or at those relevant for the radiomedical research (Cu, Ga).

## Results and discussion

### Synthesis of ligands

Previously reported synthesis of dpph utilised 1 + 1 or 2 + 2 cyclisations. The 1 + 1 cyclisation was performed with *N*,*N*′-ditosyl-2,6-bis(aminomethyl)pyridine and 2,6-bis(bromomethyl)pyridine, and the tosylated macrocycle was obtained in 55% yield.^36^ The 2 + 2 cyclisation of tosylamide and 2,6-bis(chloromethyl)pyridine yielded the tosylated macrocycle in 16 to 66%.^15,37,38^ Alternatively, the dibromo, ditosylato or dimesylato derivatives were used as alkylating agents in 2 + 2 cyclisation with tosylamide, yielding 26 to 58% of the tosylated macrocycle.^11,20,39^

**Fig. 2:**
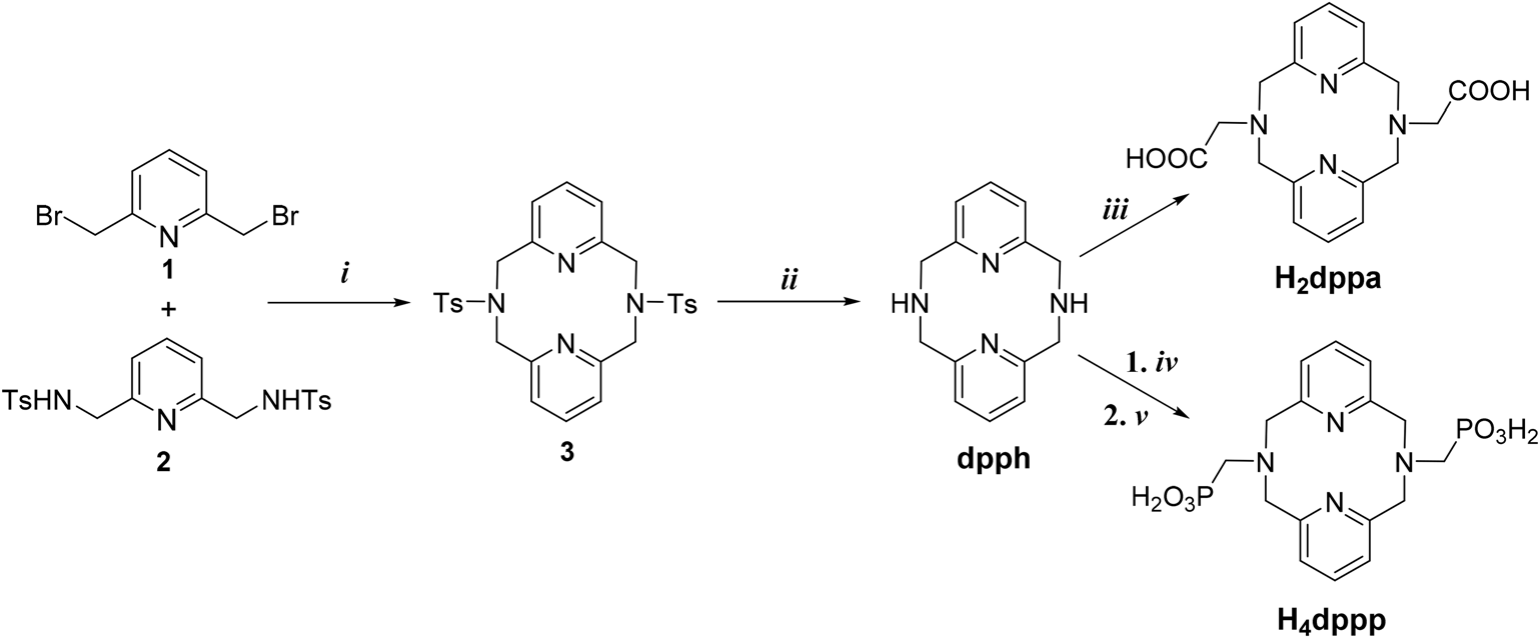
Synthesis of ligands: (i) LiOH, tetrabutylammonium iodide (catalyst), dichloromethane/water, 45 °C, 12 h; (ii) 98% H_2_SO_4_, 110 °C, 4 h; (iii) chloroacetic acid, LiOH·H_2_O, H_2_O, 60 °C, 1 day; (iv) diethylphosphite, paraformaldehyde, pyridinium hydrobromide (catalyst), pyridine, 40 °C, 1 day; (v) 6 M aq. HCl, 80 °C, 4 days.

Here, we optimized the synthesis of the tosylated macrocycle 3 by 1 + 1 cyclisation of 2,6-bis(bromomethyl)pyridine 1 and *N*,*N*′-ditosyl-2,6-bis(aminomethyl)pyridine 2 (Fig. 2). The reaction was carried out in a biphasic system using tetrabutylammonium iodide as a phase-transfer catalyst under modified conditions previously used for the synthesis of analogous tripyridinophane.^40^ The biphasic system used in this work was optimal for the cyclisation and, thus, the isolated yield (73%) was significantly higher than those reported in the works mentioned above. Removal of the tosyl groups in concentrated sulfuric acid proceeded almost quantitatively and, thus, dpph was isolated in 91% yield.

The acetate pendant arms were introduced by alkylation with chloroacetic acid.^41^ The phosphonate pendant arms were introduced by phospha-Mannich reaction with paraformaldehyde and diethylphosphite in pyridine, followed by removal of the ester groups in aqueous HCl, similarly as reported for related compounds.^42^ The reactions proceeded almost quantitatively and, thus, H_2_dppa and H_4_dppp were isolated in high yields of 92% and 83%, respectively. The NMR and MS spectra of the compounds are shown in Fig. S1–S21. The identity of H_4_dppp was also confirmed by determining its solid-state structure by X-ray diffraction analysis (Table S3 and Fig. S22). The molecular structure of H_4_dppp has a zwitterionic character – phosphonate pendant arms are mono-deprotonated, and both tertiary amino groups of the macrocycle are protonated.

### Equilibrium studies

Protonation constants of the ligands were determined by potentiometric titrations (Tables 1 and S1). The first and second constants of all ligands were found in the weakly alkaline region, and they correspond to protonations of the macrocyclic amino groups (see the solid-state structure of H_4_dppp, Fig. S22). These constants are relatively low compared to other tetraaza macrocycles due to the presence of the adjacent pyridine groups with somewhat electron-withdrawing character. Another factor is rigidity of the macrocycle with increasing number of the pyridine units. The increasing rigidity reduces formation of the intramolecular hydrogen bonds, and, consequently, also decreases basicity of the macrocycle. The role of the pyridine groups could be documented on the series of ligands listed in Table 1. Whereas the values of the corresponding constants of the cyclen derivatives are above 9, the second one is significantly lower in the pyclen derivatives, and both are lowered in the dpph derivatives. As a result, the macrocycle in the dpph derivatives is much less basic (expressed as log *K*_1_ + log *K*_2_) than in the pyclen and cyclen derivatives.

**Table 1:** Stepwise protonation constants of the title (*I* = 0.1 M (NMe_4_)Cl, 25 °C) and similar ligands

Species	dpph	H_2_dppa	H_4_dppp	Pyclen^a^	H_2_pc2a^b^	Cyclen^c^	H_2_do2a^d^
Log *K*_1_	8.04 (8.27^e^)	9.62 (9.57^f^)	9.98	10.33	12.24	10.65	11.38
Log *K*_2_	7.28 (7.36^e^)	5.89 (5.99^f^)	8.54	7.83	5.97	9.64	9.62
Log *K*_3_	—	2.27 (2.59^f^)	6.44	—	3.47	1.5	3.95
Log *K*_4_	—	1.33 (2.22^f^)	3.90	—	1.99	0.7	2.62
Log *K*_5_	—	—	0.91	—	—	—	—
Log *K*_1_ + log *K*_2_	15.32	15.51	18.52	18.16	18.21	20.29	21.00

a Ref. 44, *I* = 0.1 M KNO_3_.

b Ref. 45, *I* = 0.15 M NaCl.

c Ref. 44, *I* = 0.1 M KCl.

d Ref. 46, *I* = 0.1 M (NMe_4_)Cl.

e Ref. 16.

f Ref. 29, *I* = 0.15 M NaCl, *I* = 0.1 M KCl.

In the series of the dpph derivatives, the macrocycle basicity follows the expected order dpph < H_2_dppa < H_4_dppp.^43^ The higher basicity of the latter two is given by the negative charge of the pendant arms (double negative in the phosphonate derivative) and participation of the pendant arms in the intramolecular hydrogen bonds, which stabilize the macrocycle protonation(s). Such hydrogen bonds stabilizing the protonation state are typical for acetate and phosphonate macrocyclic ligands.^43^ The following constants of H_2_dppa and H_4_dppp belong to protonation of the pendant arms, and they are in the range expected for protonation of carboxylate and phosphonate pendant arms.^43^

The stability constants of the Ni^II^, Cu^II^, and Zn^II^ complexes were determined by means of potentiometric titrations. Whereas Cu^II^ and Zn^II^ complexation was fast, Ni^II^ complexation was slow for the conventional setup of potentiometric titrations in the acidic pH region. Thus, the stability constants of the Ni^II^ complexes were determined by the out-of-cell method with three-week-long equilibration at room temperature. The data show very low abundances of free Cu^II^ and Zn^II^ ions even at the beginning of the titrations (pH 1.7) in the systems with H_2_dppa and H_4_dppp. Thus, NMR and UV-vis titrations were performed in the strongly acidic region (pH 0–2) to determine the stability of the Zn^II^ and Cu^II^ complexes, respectively. The NMR titrations could be used to evaluate formation of the Zn^II^ complex in the strongly acidic region (Fig. S23 and S24). The free ligand and the Zn^II^ complex are in a slow exchange and, thus, separate signals of both species were found in the spectra. The ^1^H NMR signals of pyridine groups were well resolved and separated and, thus, their integrals as a function of pH were used in the combination with potentiometric data for calculation of the corresponding stability constants in the Zn^II^–H_2_dppa and Zn^II^–H_4_dppp systems. In the Zn^II^–H_4_dppp system, ^31^P NMR data were also included.

**Fig. 3:**
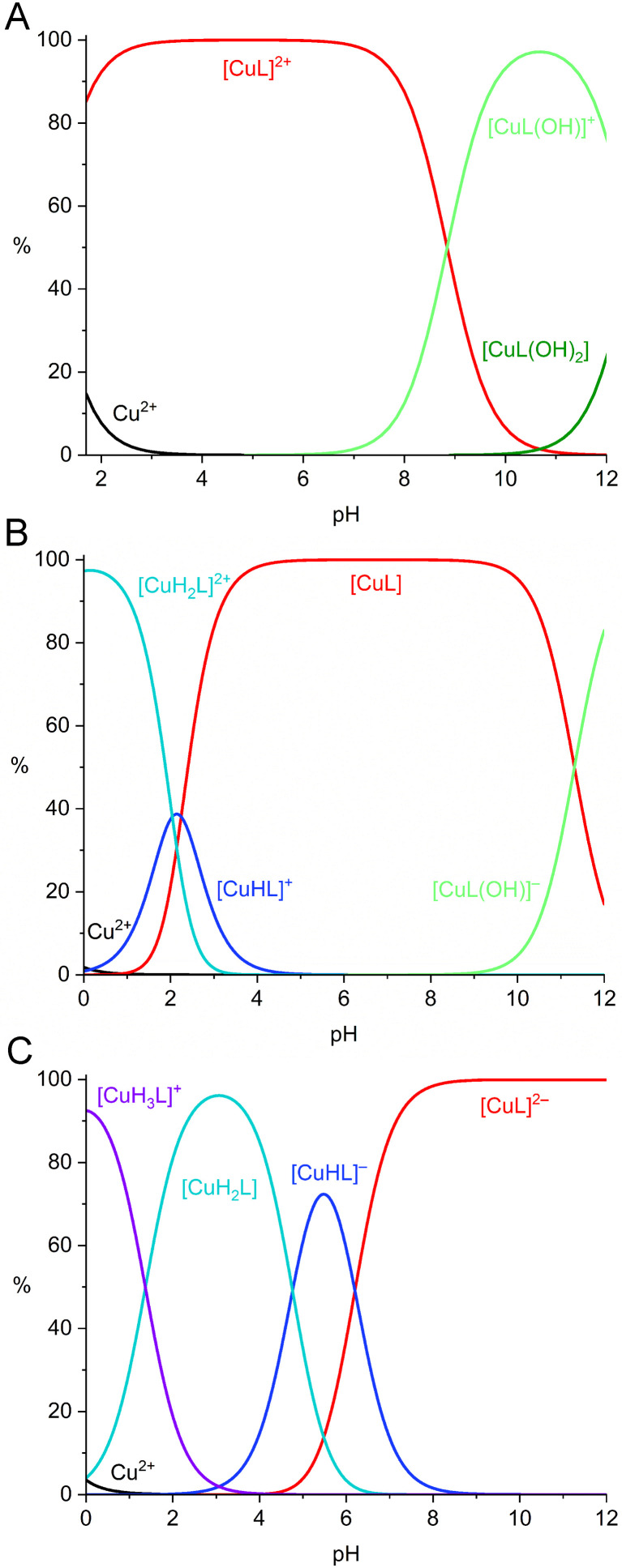
Distribution diagrams of Cu^II^–dpph (A), Cu^II^–H_2_dppa (B) and Cu^II^–H_4_dppp (C) systems (*c*_Cu_ = *c*_L_ = 0.004 m, 25 °C).

However, measurement of the UV-vis spectra in the strongly acidic region showed that Cu^II^ complexes with H_2_dppa and H_4_dppp are almost fully formed already at pH 0. Thus, the corresponding stability constants were determined by competitive UV-vis titration with 2,3,2-tet (*N*,*N*′-bis(2-aminoethyl)-1,3-propanediamine) (Fig. S25 and S26). The studied ligands and 2,3,2-tet differ significantly in basicity^44^ and, thus, Cu^II^ ion is preferentially bound to the dpph derivatives in the acidic region, whereas the coordination to 2,3,2-tet dominates in the alkaline region. Thus, the competitive titrations were performed at pH 7.7 and 7.3 for H_2_dppa and H_4_dppp, respectively, where the conditional constants of the complexes of the competing ligands are comparable (Fig. S27). In the course of titration, the initial band of the Cu^II^ complex with the corresponding dipyridinophane ligand at 840 nm gradually changes into the signal of Cu^II^-2,3,2-tet complex at 530 nm. The absorbances at both maxima as a function of pH were used in the combination with potentiometric data for calculation of the corresponding stability constants in the Cu^II^–H_2_dppa and Cu^II^–H_4_dppp systems. The spectra of the samples in the region, in which only one species is dominantly present, perfectly fit the spectra of particular complexes, that proofs negligible formation of the ternary species. The stability constants are listed in Tables 2 and S2, and the distribution diagrams are shown in Fig. 3 and S28–S33.

**Table 2:** Complex stability constants and stepwise protonation constants for complexes of the studied ligands (*I* = 0.1 M (NMe_4_)Cl, 25 °C)

Ligand	Equilibrium	Ni^II^	Cu^II^	Zn^II^
dpph	M + L ⇄ [M(L)]	14.54	15.98	13.09
	[M(L)(OH)] + H^+^ ⇄ [M(L)]	11.36	8.88	9.60
	[M(L)(OH)_2_] + H^+^ ⇄ [M(L)(OH)]	—	12.57	—
H_2_dppa	M + L ⇄ [M(L)]	14.29	20.48	17.50
	[M(L)] + H^+^ ⇄ [M(HL)]	2.83	2.24	2.35
	[M(HL)] + H^+^ ⇄ [M(H_2_L)]	—	2.04	—
	[M(L)(OH)] + H^+^ ⇄ [M(L)]	—	11.32	12.30
H_4_dppp	M + L ⇄ [M(L)]	17.51	22.64	18.93
	[M(L)] + H^+^ ⇄ [M(HL)]	6.46	6.20	5.80
	[M(HL)] + H^+^ ⇄ [M(H_2_L)]	4.96	4.76	4.45
	[M(H_2_L)] + H^+^ ⇄ [M(H_3_L)]	—	1.37	—

Dpph does not form protonated complexes due to the absence of the pendant arms. Thus, the [ML] species dominate in the acidic region in all systems. As the ligand does not saturate the metal ion coordination sphere, hydroxido complexes are formed in the alkaline region. H_2_dppa complexation starts with the formation of protonated complexes in the acidic region. Monoprotonated species are dominant at the beginning of titration in the systems with Zn^II^ and Ni^II^ ions, whereas diprotonated species were found in the strongly acidic solutions in the Cu^II^–H_2_dppa system. Corresponding protonation constants are close to those found for carboxylates in the free ligand, which indicates only a weak (or negligible) coordination of the pendant arms. In all H_2_dppa systems, [ML] is the dominant species over a broad pH region (pH 3–11), and the hydroxido complexes were found only in the strongly alkaline solutions. H_4_dppp forms protonated complexes along the whole acidic region. The first and the second protonation constants of the complexes (log *K*_1_ = 5.8–6.5, log *K*_2_ = 4.4–5.0) are in the common range reported for phosphonate groups in macrocyclic complexes.^43^ Similarly to the Cu^II^–H_2_dppa system, additional protonation was also found in the Cu^II^–H_4_dppp system and, thus, the triprotonated complex is the dominant species in the strongly acidic region. The stability constants of the dpph complexes follow the order Cu^II^ > Ni^II^ > Zn^II^, whereas the stability constants of the Ni^II^ and Zn^II^ complexes are reversed for H_2_dppa and H_4_dppp, which can be explained by the high oxophilicity of the Zn^II^ ion.

The comparison with related cyclen/pyclen-based ligands (Table 3) shows that the stability constants correlate with the macrocycle basicity. Thus, the stabilities of the complexes follow the order dpph < pyclen < cyclen. The same order is also observed for complexes of the acetate derivatives (H_2_dppa < H_2_pc2a < H_2_do2a), whose stability constants are mostly higher than those of the parent macrocycles due to additional coordination of the pendant arms. Stabilities of Ni^II^ complexes are somewhat out of the trends observed for other metal ions – [Ni(dppa)] being less stable than [Ni(dpph)]. It could be rationalized by labile coordination of the carboxylate groups of H_2_dppa, which was also observed in the solid state (see lower).

**Table 3:** Stability constants log *K*_ML_ and free metal concentration pM (*c*_M_ = 0.1 mM, *c*_L_ = 1 mM, pH 7.4) of the studied complexes (*I* = 0.1 M (NMe_4_)Cl, 25 °C) and of complexes of related ligands

Metal ion	dpph		H_2_dppa		H_4_dppp		Pyclen^a^		H_2_pc2a^b^		Cyclen^c^		H_2_do2a^d^	
	Log *K*_ML_	pM	Log *K*_ML_	pM	Log *K*_ML_	pM	Log *K*_ML_	pM	Log *K*_ML_	pM	Log *K*_ML_	pM	Log *K*_ML_	pM
Ni^II^	14.54	14.6	14.29	13.0	17.51	14.7	17.05	14.5	—	—	16.4	11.9	—	—
Cu^II^	15.98	16.0	20.48	19.2	22.64	19.8	20.14	17.6	23.58	19.7	24.6	20.1	24.24	19.0
Zn^II^	13.09	13.1	17.50	16.2	18.93	16.1	14.40	11.9	19.49	15.6	16.2	11.7	18.86	13.6

a Ref. 44, *I* = 0.1 M KNO_3_.

b Ref. 45, *I* = 0.15 M NaCl.

c Ref. 44, *I* = 0.1 M KCl.

d Ref. 46, *I* = 0.1 M (NMe_4_)Cl.

As stability constants of the complexes typically correlate with protonation constants of the ligands, chelating abilities of the ligands for biomedical applications are often evaluated in the terms of pM values, *i.e.* negative logarithm of the free metal ion concentration at physiological pH. Thus, corresponding pM values of the discussed complexes were calculated and they are shown in Table 3. It is evident that chelating ability of the dipyridinophane ligands at physiological pH is comparable or even higher than those of the pyclen, cyclen and their diacetate derivatives and it could be clearly ascribed to the decreased basicity of macrocyclic nitrogen atoms in the dipyridinophane derivatives.

### Complexes in the solid state

Seven complexes of the studied ligands with divalent metal ions – isostructural complexes [M(dpph)Cl_2_]·H_2_O·0.5*i*PrOH (M = Ni, Cu, Zn), [Ni(dppa)(H_2_O)_2_]·2H_2_O, isostructural [M(dppa)]·2H_2_O (M = Ni, Cu), and {[Cu(H_2_dppp)]_2_}·10H_2_O·2CH_3_COCH_3_ were prepared in the form of single crystals and their structures were determined by X-ray diffraction. In addition, three complexes with trivalent metal ions – [Co(dppa)]Cl·4.5H_2_O, [Fe(dppa)Cl]·4H_2_O, and {[Ga(dppa)]_4_}Cl_4_·17H_2_O – were also structurally characterized. When discussing these crystal structures in the following text, only the complex unit is stated for simplicity, without water/acetone molecules of crystallisation or counterions.

The complexes were prepared by a reaction of the ligand with the corresponding metal chloride, except the Co^III^ complex, which was formed by a reaction with CoCl_2_. Preparation of Co^III^ complex was performed in the presence of air during complexation and crystallization, which enabled oxidation of Co^II^ to Co^III^. The crystallographic data are summarised in Table S3, and selected geometric parameters of the metal ions' coordination spheres are listed in Table S4.

**Fig. 4:**
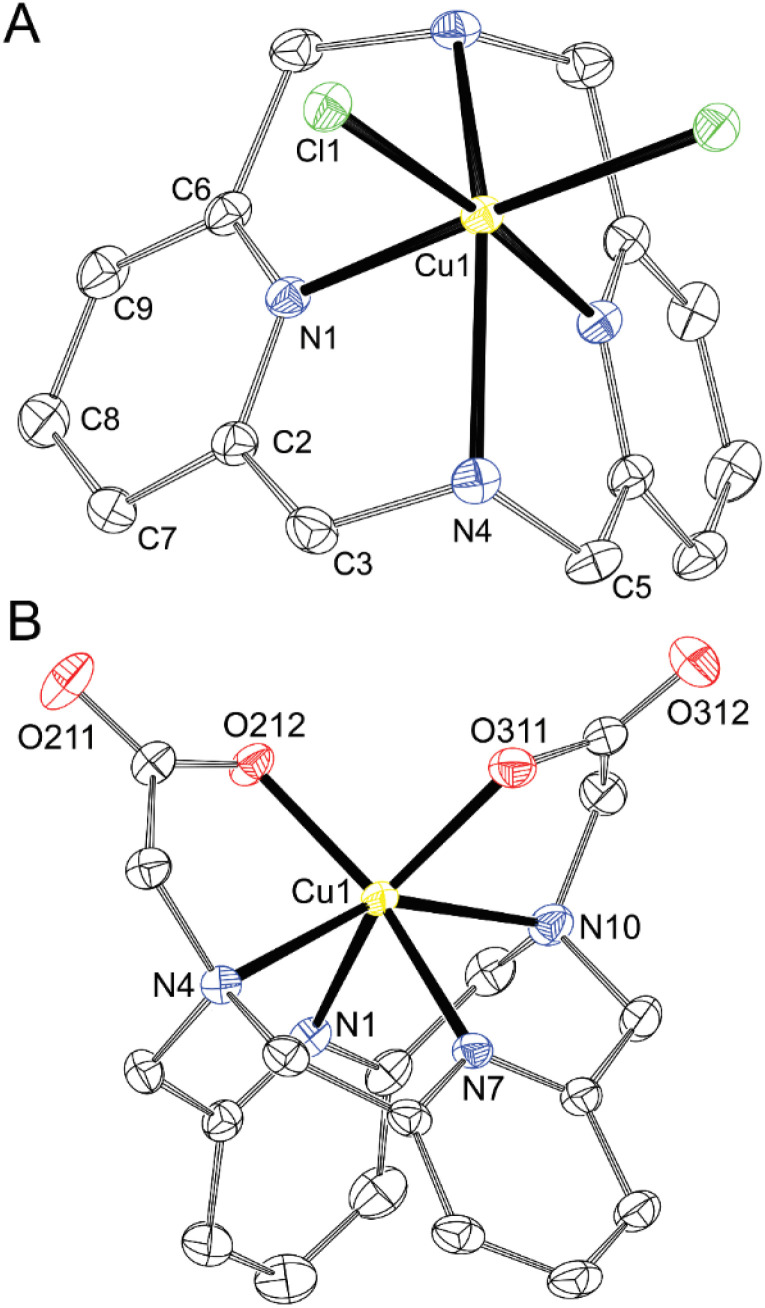
Molecular structures of [Cu(dpph)Cl_2_] found in the crystal structure of [Cu(dpph)Cl_2_]·H_2_O·0.5*i*PrOH (A, ∢*κ* = 176°, ∢N4–Cu1–N4# = 148°) and of [Cu(dppa)] found in the crystal structure of [Cu(dppa)]·2H_2_O (B, ∢*κ* = 174 and 156°, ∢N4–Cu1–N10 = 148°). The hydrogen atoms are not shown for clarity. Thermal ellipsoids are drawn at 50%.

**Fig. 5:**
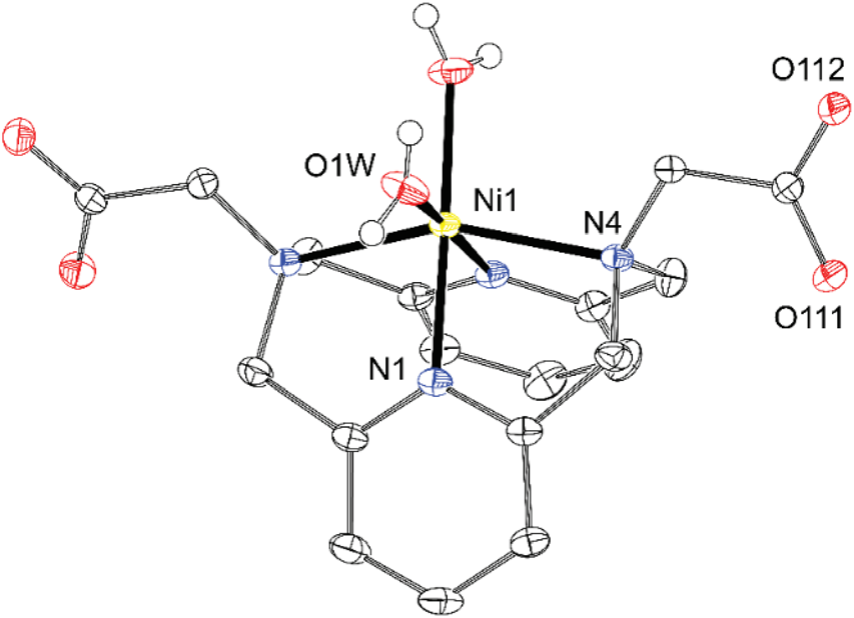
Molecular structure of [Ni(dppa)(H_2_O)_2_] found in the crystal structure of [Ni(dppa)(H_2_O)_2_]·2H_2_O (∢*κ* = 169°, ∢N4–Cu1–N4# = 154°). The carbon-bound hydrogen atoms are not shown for clarity. Thermal ellipsoids are drawn at 50%.

Metal ions in all divalent metal complexes exhibit a distorted octahedral coordination sphere in which the two pyridine moieties are coordinated in mutual *cis* positions, and the two aliphatic amino groups are bound *trans* to each other. The complexes [Cu(dpph)Cl_2_], [Ni(dpph)Cl_2_] and [Zn(dpph)Cl_2_], where the remaining two positions are occupied by chloride anions compensating the positive charge of the metal ion, have crystallographical *C*_2_-symmetry and their geometries are very similar due to isostructurality of [M(dpph)Cl_2_]·H_2_O·0.5*i*PrOH phases (Tables S3 and S4). Therefore, only one representative example is shown in Fig. 4A. The crystal structures of the complexes [Cu(dpph)Cl_2_] and [Zn(dpph)Cl_2_] with essentially the same lattice parameters (and also very similar geometries of the complex molecules) have already been deposited in the Cambridge Structural Database by others, but the disordered solvate molecules in these structures were treated using SQUEEZE.^12,20^ Therefore, we present the newly determined crystal structures here, as we successfully modelled the disorder of the solvent molecules of crystallisation. It should be noted that another crystalline phase has already been reported for the NiCl_2_–dpph system, [Ni_2_(dpph)_2_(µ-Cl)_2_]Cl_2_·2MeOH, where two [Ni(dpph)] complexes having a symmetry plane are connected through two bridging chloride ions into a centrosymmetric dimer.^12,19^ In the isostructural [Cu(dppa)] and [Ni(dppa)] complexes, the remaining sites of the octahedral coordination environment are occupied by carboxylate oxygen atoms of the pendant arms (Fig. 4B). The complex molecules have rough (not crystallographic) *C*_2_-symmetry, which is disrupted by different tilting of the pyridine rings (see below). In addition, another form of Ni^II^ complex of the composition [Ni(dppa)(H_2_O)_2_] was isolated (light purple needles compared to deep purple prisms of [Ni(dppa)]). In this complex, the coordination sphere is completed with two water molecules, and the carboxylate groups are not coordinated (Fig. 5). This fact is somewhat surprising as the carboxylic acid pendant arms are deprotonated, but do not form the chelate rings. The difference in the position of the metal ion in the macrocyclic cavity of both Ni^II^ complexes is negligible as could be documented on N–Ni–N distances and angles (Tables 4 and S4). In addition, the differences between metal ions in the isostructural complexes [M(dpph)Cl_2_] are bigger than difference between both forms of [Ni(dppa)] complex.

**Table 4:** The angle *κ* between the pyridine ring and the pyridine–metal coordination bond; the angle between coordination bonds to both amine groups (N–M–N angle)

Complex	∢*κ* (°)	∢N–M–M (°)	Complex	∢*κ* (°)	∢N–M–M (°)
[Ni(dpph)Cl_2_]	174.91(6)	152.13(6)	[Cu(dppa)]	174.18(7)/155.85(7)	148.40(4)
[Ni_2_(dpph)_2_(µ-Cl)_2_]^2+^	175.4/179.0^a^	152–153	[Cu(H_2_dppa)Cl_2_] c^,^^d^	163.0/178.2	147–148
	175.6/178.7^b^			175.0/177.1	
[Cu(dpph)Cl_2_]	175.71(6); 176.5^e^	147.65(6)	{[Cu(Hdppa)]_4_}^4+^^c^	158.7/176.5	147.26
[Zn(dpph)Cl_2_]	175.38(6); 173.2 a	144.61(6)	[Co(dppa)]^+^	179.59(8)/178.83(9)	167.95(6)
[Ni(dppa)]	171.03(5)/157.91(6)	155.53(4)	[Fe(dppa)Cl]	179.14(9)/176.83(9)	138.85(6)
[Ni(dppa)(H_2_O)_2_]	169.40(5)	153.88(5)	[Ga(dppa)]^+^^d^	176.40(8)/172.45(9)	143.09(6)
			[Cu(H_2_dppp)]^+^	172.87(9)/171.05(8)	145.42(5)
				169.95(6)/165.84(6)	149.85(4)

a Ref. 12.

b Ref. 19.

c Ref. 31.

d Two independent complex molecules.

e Ref. 20.

**Fig. 6:**
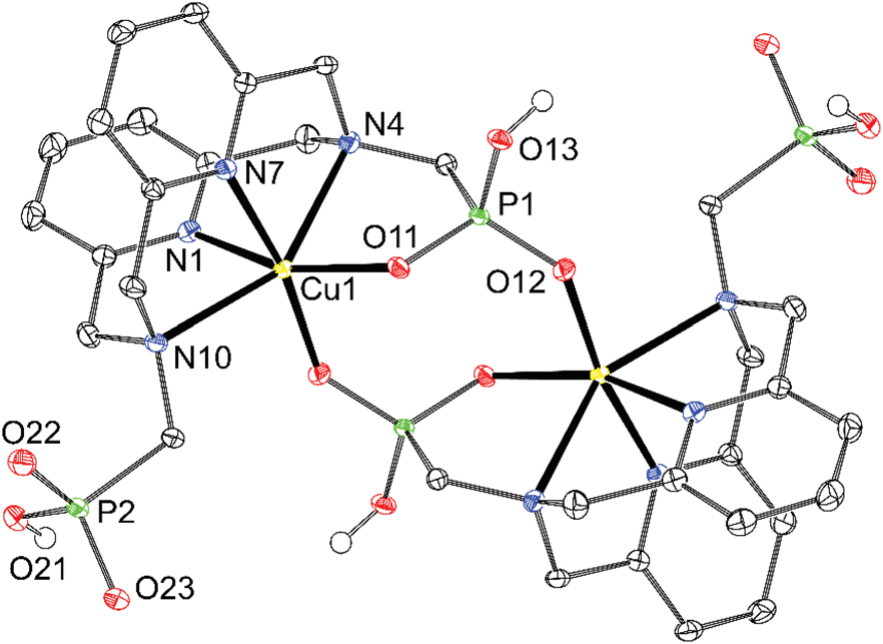
Molecular structure of [Cu(H_2_dppp)]_2_ dimeric unit found in the crystal structure of {[Cu(H_2_dppp)]_2_}·10H_2_O·2CH_3_COCH_3_ (∢*κ* = 166 and 170°, ∢N4–Cu1–N10 = 150°). The carbon-bound hydrogen atoms are not shown for clarity. Thermal ellipsoids are drawn at 50%.

Both phosphonate groups in the [Cu(H_2_dppp)] complex are monoprotonated, but only one of them is coordinated. The other phosphonate remains uncoordinated, and the last coordination site of the distorted octahedron is occupied by an oxygen atom of the phosphonate group, which is coordinated to the Cu^II^ ion in the neighbouring (centrosymmetry-related) complex unit (Fig. 6). The metal ions and the phosphonate groups of the bridging unit form an eight-membered metalla-ring, which is common for phosphonate complexes.^47^

To document the extent of a macrocycle geometric strain, we examined the angle between the idealised (mean) pyridine plane and the pyridine-metal coordination bond *κ* (Table 4). In the relaxed structures of dpph complexes, the angle is almost straight (*κ* = 175–176°), *i.e.*, the metal ion lies in the direction of the lone electron pair of the nitrogen atom. This finding is consistent with the previously reported crystal structures.^12,19,20^ On the contrary, one of these angles is significantly bent (*κ* = 156–158°) in the strained structures of H_2_dppa complexes in which both pendant arms are coordinated. It confirms the steric strain of H_2_dppa when hexacoordinated to the Cu^II^ or Ni^II^ ions. Similar significant bending of one of the pyridine groups was previously observed in the crystal structure of {[Cu(Hdppa)]_4_}(ClO_4_)_4_·9H_2_O·3MeCN (*κ* = 159° and 177°),^31^ in which one of the pendant arms is protonated and only semi-coordinated to the central metal ion. However, this structure is complicated by the bridging of neighbouring units through the deprotonated acetate group into a cyclotetramer, which deforms the coordination environment. Decoordination of the pendant arms leads to a more relaxed geometry of the macrocycle in [Ni(dppa)(H_2_O)_2_] (*κ* = 169°), which is closer to the [M(dpph)Cl_2_] complexes. Analogous geometry was previously reported for the solid-state structure of [Cu(H_2_dppa)Cl_2_] with protonated and uncoordinated carboxylate groups (*κ* = 163/178° and 175/177° for two independent units, respectively).^31^ In the [Cu(H_2_dppp)] complex, coordination of only one pendant arm likely does not lead to a high geometric strain and, thus, the pyridine coordination angles are relatively straight (*κ* = 166° and 170°) when compared with the complexes of hexacoordinated H_2_dppa.

**Fig. 7:**
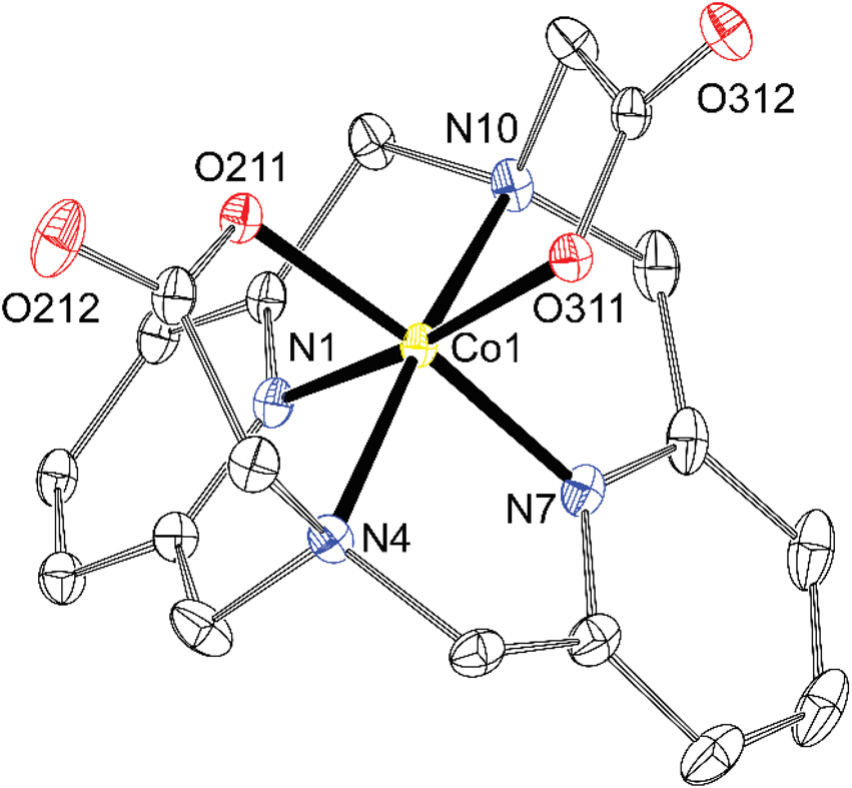
Molecular structure of the [Co(dppa)]^+^ cation found in the crystal structure of [Co(dppa)]Cl·4.5H_2_O (∢*κ* = 179 and 180°, ∢N4–Cu1–N10 = 168°). The hydrogen atoms are not shown for clarity. Thermal ellipsoids are drawn at 50%.

**Fig. 8:**
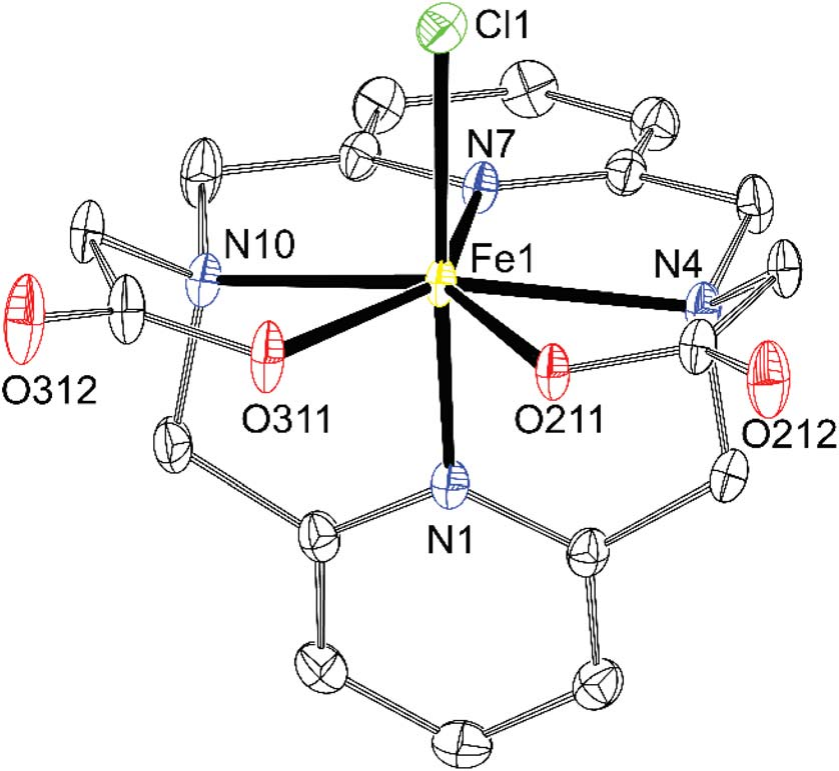
Molecular structure of [Fe(dppa)Cl] found in the crystal structure of [Fe(dppa)Cl]·4H_2_O (∢*κ* = 177 and 179°, ∢N4–Cu1–N10 = 134°). The hydrogen atoms are not shown for clarity. Thermal ellipsoids are drawn at 50%.

**Fig. 9:**
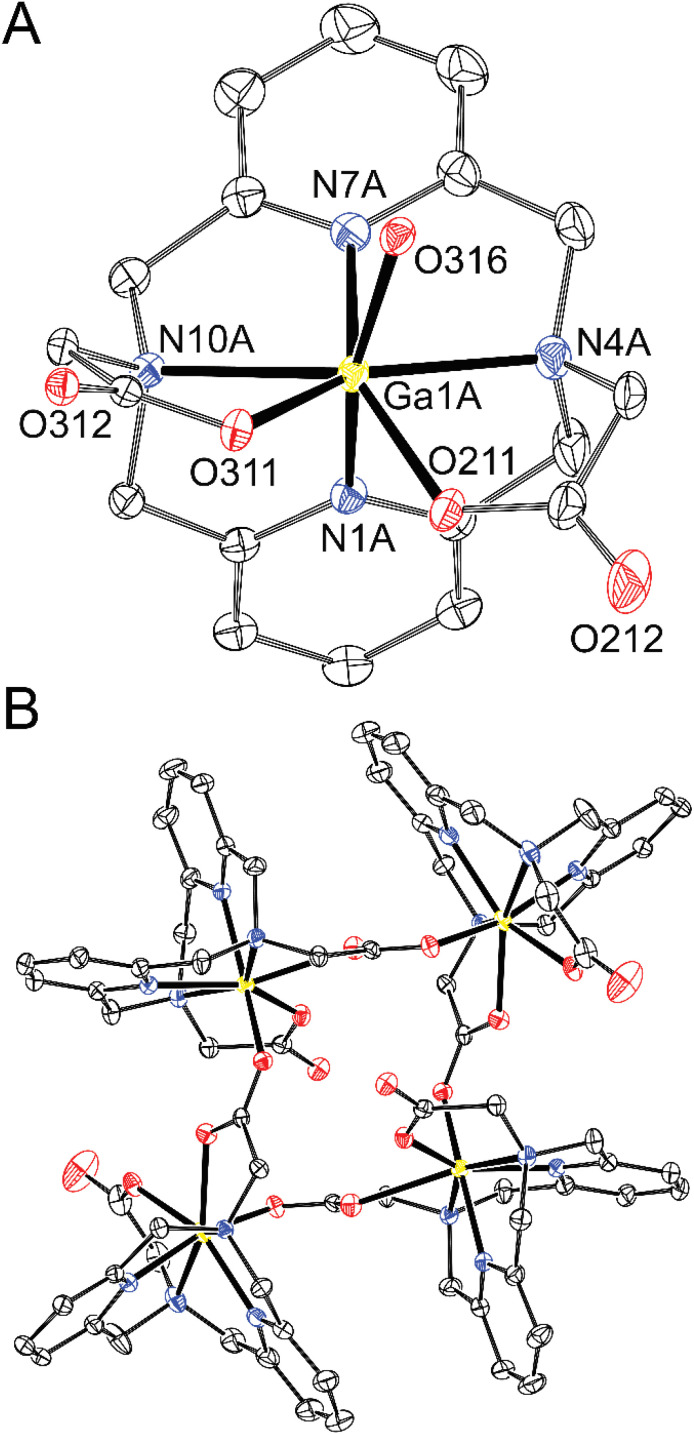
Molecular structure of one of two independent [Ga(dppa)]^+^ cations (A) and of the {[Ga(dppa)]_4_}^4+^ tetramer (B) found in the crystal structure of {[Ga(dppa)]_4_}Cl_4_·17H_2_O (∢*κ* = 171–176°, ∢N4–Cu1–N10 = 143 and 145°). The hydrogen atoms are not shown for clarity. Thermal ellipsoids are drawn at 50%.

Exploring values of the *trans* angle N(amine)–M–N(amine), which is far from theoretical 180° (145–152°, Table S4), indicates that the divalent first-row transition metal ions (*r* = 83–88 pm)^48^ are too large for the cavity formed by hexacoordinated dipyridinophane. Complexes of trivalent metal ions behave differently. The Co^III^ ion fits the octahedral coordination environment defined by the dppa^2−^ cavity much better due to its small radius (69 pm) as documented by the *trans* angle of 169° and almost straight coordination of the pyridine fragments (*κ* = 179°) in the [Co(dppa)]^+^ species (Fig. 7). However, for other trivalent metal ions Fe^III^ and Ga^III^, heptacoordination was observed due to interaction of the [M(dppa)]^+^ species with the chloride ion (Fe^III^) or neighbouring complex molecules (Ga^III^). As more space is required for the additional three coordinated atoms over the M–dpph fragment, the *trans* N–M–N angle is bent 139–145° (Table S4), but the pyridine coordination angle is maintained straight in these complexes (*κ* = 177–179° and 171–176°, for [Fe(dppa)Cl] and [Ga(dppa)]^+^, respectively). The coordination sphere of [Fe(dppa)Cl] is close to a pentagonal bipyramid in which one pyridine is coordinated in the axial position and all other donor atoms of dppa^2−^ form the equatorial pentagon N_3_O_2_. The second axial position is occupied by a chloride anion (Fig. 8). Pentagonal bipyramide has been commonly found in the crystal structures of the Fe^III^ complexes,^28^ also with several examples ofapical chloride coordination.^49–52^ The coordination sphere of [Ga(dppa)]^+^ can be viewed similarly, but a carboxylate oxygen atom from the neighbouring complex unit is bound in the second apical position. Such a connection forms a cyclotetrameric structure [Ga(dppa)]_4_^4+^ (Fig. 9). Also, the pentagonal base is more distorted from the ideal plane in this case. Heptacoordination of Ga^III^ is less usual, and was observed, *e.g.*, for mixed phenanthroline–carboxylato complex,^53^ or for GaCl_2_^+^ fragment complexed by 15-crown-5,^54^ where almost a regular pentagonal bipyramide with apical coordination of the chloride ions was observed. However, the pentagonally bipyramidal N–N_3_O_2_–O coordination sphere reported here is the first example. The tetrameric structural motif resembles that found previously in {[Cu(Hdppa)]_4_}(ClO_4_)_4_·9H_2_O·3MeCN,^31^ in which one carboxylate of each ligand bridges the tetrameric complex, whilst the other pendant arm is protonated and semi-coordinated. The heptacoordination and similar geometries of Fe^III^ and Ga^III^ complexes indicate that the [Fe(dppa)Cl] complex has a high-spin arrangement, as Ga^III^ and high-spin Fe^III^ ions have very similar ionic radii (76 and 78.5 ppm, respectively).

## Conclusions

The pyridine rings in the studied dipyridinophane ligands decrease the basicity of the macrocycle and, thus, the basicities of tetraazamacrocycles follow the order dipyridinophane < pyclen < cyclen. Consequently, the same order is observed for stability constants. However, the low macrocycle basicity of dipyridinophane ligands bearing two coordinating pendant arms is responsible for their high complexing ability in highly acidic solutions – Cu^II^ complexes with H_2_dppa and H_4_dppp are formed almost quantitatively even at pH 0. It is somewhat surprising considering the solid-state structures of complexes with divalent metal ions isolated from acidic solutions in which pendant arms are often protonated and uncoordinated. The octahedral geometry is quite distorted. The pyridine units are coordinated in mutual *cis* positions, and the pyridine coordination angle is bent. The macrocycle amino groups occupy *trans* positions, and the corresponding *trans* N–M–N angle is significantly lower than the ideal 180°. The pendant arms are uncoordinated in some structures. It indicates that the geometry of dipyridinophane ligands with two coordinating pendant arms is not optimal for the complexation of divalent metal ions. On the contrary, trivalent metal ions fit perfectly into the cavity formed by dppa^2–^. It shows that dipyridinophane ligands are quite sensitive towards the radius and steric requirements of particular metal ions as a result of the macrocycle rigidity. The structural data indicate that dipyridinophane is a suitable ligand for very small metal ions (*r* < 70 pm) which prefer octahedral coordination, or medium-size metal ions (*r* 75–80 pm) preferring pentagonal bipyramidal complexes. The geometric preferences and the high coordination ability in the acidic solutions are important outcomes for future design of metal ions' carriers in molecular imaging, radiotherapy and other fields of bioinorganic chemistry.

## Experimental

### General

All commercially available starting materials (Fluka, Aldrich, Chematech, Lachema, Fluorochem, Apollo Scientific) were used without further purification. 2,6-bis(bromomethyl)pyridine (1) and 2,6-bis{[(4-methylphenyl)sulfonamido]methyl}pyridine (2) were synthesised according to the published procedures (detailed procedures are described in SI).^39,55,56^

The NMR spectra were acquired at 25 °C on a Bruker 400 Neo or Varian S300 spectrometers. The experiments in non-deuterated water were acquired without water signal suppression. For ^1^H and ^13^C{^1^H} NMR measurements, the methyl signal of *t*BuOH was used as an internal standard (*δ* 1.25 and 30.3 ppm for ^1^H and ^13^C{^1^H} NMR, respectively); ^31^P NMR spectra were referenced to external 85% H_3_PO_4_ (*δ* 0.0 ppm). All values of chemical shifts are given in ppm, and coupling constants are given in Hz. Electrospray ionisation mass spectra (ESI-MS) and analytical HPLC-MS measurements were performed using a Waters Acquity QDa instrument equipped with dual orthogonal electrospray ionisation (ESI) at atmospheric pressure and a quadrupole mass analyser operating in the *m*/*z* range of 30–1250. HRMS measurements were performed on Orbitrap LTX XL Thermo. The UV-vis spectra were recorded on a Specord 50 Plus system (Analytic Jena).

The HPLC analyses were conducted with a silica-based reverse-phase column (Phenomenex Luna C18, 5.0 µm, 4.6 × 50 mm; dead time 0.5 min), using mobile phases 0.1% trifluoroacetic acid (TFA) in H_2_O and 0.1% TFA in acetonitrile (MeCN), applied with gradient: 0.0 min – 5% MeCN, 3.5 min – 100% MeCN, 4.0 min – 100% MeCN, 5.0 min – 5% MeCN (flow 1.2 ml min^−1^). Preparative chromatographic separations (“flash” chromatography) were carried out using an ECOM ECS28P0X system equipped with a UV-vis diode array detector (*λ* = 200–800 nm). Reverse-phase separation was performed using 40 or 120 g pre-packed columns (Phenomenex Luna C18, 5.0 µm) and a gradient elution with MeCN (5% to 100% in 40 min) in water with 0.1% TFA at a flow rate of 40 ml min^−1^.

### Synthesis

#### 3,7-Bis[(4-methylphenyl)sulfonyl]-3,7-diaza-1,5(2,6)-dipyridinacyclooctaphane (3)

The refluxing biphasic mixture of tetrabutylammonium iodide (2.49 g, 6.7 mmol) and 2 (5.00 g, 11.0 mmol) in dichloromethane (150 ml) and LiOH·H_2_O (23.5 g, 560 mmol) in distilled water (120 ml) was vigorously stirred. A solution of 1 (2.97 g, 11.0 mmol, 1.0 equiv.) in dichloromethane (75 ml) was added dropwise over 3 h. The stirred reaction mixture was refluxed for additional 24 h. After cooling to room temperature, the organic phase was separated and dried with anhydrous MgSO_4_. The solids were filtered off, and volatiles were removed under reduced pressure. The residue was re-dissolved in chloroform (15 ml), and MeOH (150 ml) was added to induce precipitation. The resulting solid was collected by filtration through a fine glass frit, affording a yellowish powder. The final purification was achieved by recrystallisation from hot chloroform, yielding the product as a white powder (4.50 g, 73%). ^1^H NMR (CDCl_3_): 7.79 (d, ^3^*J*_HH_ 7.9, 4H, Ts), 7.38 (m, 6H, py + Ts), 7.18 (d, ^3^*J*_HH_ 7.7, 4H, py), 4.50 (s, 8H, CH_2_), 2.46 (s, 6H, Ts). ^13^C{^1^H} NMR (CDCl_3_): 154.8 (s, py), 143.8 (s, Ts), 137.4 (s, Ts), 135.9 (s, py), 130.0 (s, Ts), 127.0 (s, Ts), 123.2 (s, Py), 56.4 (s, CH_2_), 21.6 (s, CH_3_). MS(+): *m*/*z* 549.2 [M + H]^+^, HRMS(+): 549.1617 (calc. 549.1630) [M + H]^+^.

#### 3,7-Diaza-1,5(2,6)-dipyridinacyclooctaphane (dpph)

The tosylated pyridinophane 3 (4.50 g, 8.2 mmol) was dissolved in concentrated sulfuric acid (98%, 50 ml). The reaction mixture was stirred at 115 °C for 2 h. Then, the solution was cooled to room temperature, poured onto crushed ice (200 ml), and neutralised by slow addition of 20% aq. NaOH solution until it became alkaline (pH 12). The resulting aqueous mixture was extracted with chloroform (3 × 100 ml). The combined organic phases were dried with anhydrous MgSO_4_, filtered, and concentrated under reduced pressure to yield the product as a white powder (1.80 g, 91%).

^1^H NMR (CDCl_3_): 7.07 (t, ^3^*J*_HH_ 7.6, 2H, py), 6.51 (d, ^3^*J*_HH_ 7.6, 4H, py), 4.45 (s, 2H), 4.00 (s, 8H, CH_2_). ^13^C{^1^H} NMR (CDCl_3_): 159.1 (s, py), 135.8 (s, Py), 120.05 (s, py), 55.9 (s, CH_2_). MS(+): *m*/*z* 241.1 [M + H]^+^. Elemental analysis: found (calculated for C_14_H_16_N_4_·0.5H_2_O): C 67.37 (67.59), H 6.70 (6.88), N 22.16 (22.45).

#### (3,7-Diaza-1,5(2,6)-dipyridinacyclooctaphane-3,7-diyl)-diacetic acid (H_2_dppa)

Dpph (0.50 g, 2.1 mmol) was dissolved in deionised water (40 ml), and chloroacetic acid (0.98 g, 10 mmol) and LiOH·H_2_O (1.05 g, 25 mmol) were added. The reaction mixture was stirred at 60 °C for 24 h. Reaction progress was followed by HPLC. After cooling to room temperature, the solution was purified on a cation exchange column (Dowex 50). Impurities were eluted with water, while the product was eluted with 10% aq. pyridine. Volatiles were removed under reduced pressure, and pyridine residue was removed by repeated co-evaporation with distilled water. The final product was obtained as a light-yellow powder (0.68 g, 92%).

^1^H NMR (D_2_O/NaOH, pH 13.0): 7.21 (t, ^3^*J*_HH_ 7.7, 2H, py), 6.67 (d, ^3^*J*_HH_ 7.7, 4H, py), 3.81 (s, 8H, py–CH̲_2_–N), 3.47 (s, 4H, CH̲_2_COO). ^13^C{^1^H} NMR (D_2_O/NaOH, pH 13.0): 180.3 (s, COO), 158.5 (s, py), 137.9 (s, py), 122.4 (s, Py), 64.0 (s, C̲H_2_COO), 63.8 (s, py–C̲H_2_–N). MS(+): *m*/*z* 357.1 [M + H]^+^. HPLC: 2.11 min. Elemental analysis: found (calculated for C_18_H_20_N_4_O_6_): C 60.32 (60.66), H 5.42 (5.66), N 15.35 (15.72).

#### (3,7-Diaza-1,5(2,6)-dipyridinacyclooctaphane-3,7-diyl)-bis(methylenephosphonic acid) (H_4_dppp)

Dpph (0.30 g, 1.25 mmol) was dissolved in anhydrous pyridine (7 ml) in a 10 ml round-bottom flask. Pyridinium hydrobromide (0.40 g, 2.5 mmol), diethyl phosphite (0.64 ml, 0.69 g, 5.0 mmol), and a small amount of anhydrous MgSO_4_ (20 mg) were added. Subsequently, paraformaldehyde (0.15 g, 5.0 mmol) was added, and the reaction mixture was stirred at 40 °C for 24 h. The conversion of the reaction was monitored by ^31^P NMR spectroscopy. After completion, the mixture was filtered through a fine glass frit and the filtrate was evaporated under reduced pressure. The resulting oily residue was co-evaporated with toluene (5 ml) four times to remove residual pyridine. The remaining oil was dissolved in acetonitrile and purified by reverse-phase flash chromatography. The product-containing fractions were combined and evaporated. The residue was then dissolved in 6 M aq. HCl (20 ml), and the solution was heated to 80 °C for 4 days to achieve complete de-esterification of the ethyl groups. Solvents were removed under reduced pressure, and the residue was further co-evaporated three times with water to eliminate any residual HCl. The crude product was purified by cation exchange chromatography (Dowex 50). Impurities were eluted with water, and the product was eluted with 10% aq. pyridine. Volatiles were removed under reduced pressure, and pyridine residue was removed by repeated co-evaporation with distilled water. The product was obtained as a white powder (0.45 g, 85%).

^1^H NMR (D_2_O/DCl, pH 0.8): 7.75 (t, ^3^*J*_HH_ 7.8, 2H, py), 7.27 (d, ^3^*J*_HH_ 7.8, 4H, py), 4.82 (s, 8H, py–CH̲_2_–N), 3.90 (d, ^2^*J*_PH_ 11.5, 4H, N–CH̲_2_–P). ^13^C{^1^H} NMR (D_2_O/DCl, pH 0.8): *δ* 151.0 (s, py), 142.3 (s, py), 125.0 (s, py), 61.7 (d, ^3^*J*_CP_ 4.4 Hz, py–C̲H_2_–N), 55.8 (d, ^1^*J*_CP_ 142 Hz, N–C̲H_2_–P). ^31^P NMR (D_2_O/DCl, pH 0.8): 11.73 (t, ^3^*J*_PH_ 11.5 Hz)*.* HPLC: 1.32 min. MS(+): *m*/*z* 429.1 [M + H]^+^. Elemental analysis: found (calculated for C_16_H_22_N_4_O_6_P_2_·1.5H_2_O): C 42.38 (42.20), H 5.31 (5.53), N 12.23 (12.30), P 14.06 (13.60).

### Potentiometry

Potentiometry was carried out according to previously published procedures; for the preparation of stock solutions and chemicals, equipment, electrode system calibration, titration procedures and data treatment, see ref. 57. Throughout the paper, pH means −log[H^+^]. Protonation constants (*c*_L_ = 0.004 m) and stability constants of Cu^II^ and Zn^II^ complexes (*c*_L_ = *c*_M_ = 0.004 m) were determined in 0.1 M (NMe_4_)Cl at 25.0 °C with p*K*_w_ = 13.81 by in-cell titrations from data obtained in the pH range 1.6–12 with ∼40 points per titration and four parallel titrations. The stability constants of the Ni^II^ complexes were obtained by the out-of-cell method as described previously^58^ in the pH range 1.7–6 with ∼20 points per titration and four parallel titrations. The constants were calculated with the OPIUM program.^59^ Stability constants of metal ion hydroxido complexes were taken from ref. 44.

### NMR titration of Zn^II^–H_2_dppa and Zn^II^–H_4_dppp systems in strongly acidic solution

The samples were prepared with the same concentrations used for potentiometry (*c*_L_ = *c*_M_ = 0.004 m, formal pH range 0.0–1.5, 15 samples) using 0.2 M or 1 M aq. HCl stock solutions to get the final sample volume of 1 ml. Ionic strength was not strictly controlled. Stability constants of the Zn^II^ complexes were determined by simultaneous treatment of the potentiometric and NMR data (^1^H and ^31^P integral intensities of signals of the free ligand and the complex as a function of pH) using the OPIUM program.^59^

### UV-vis competition titration of Cu^II^–H_2_dppa and Cu^II^–H_4_dppp systems

The samples were prepared with the same concentrations used for potentiometry (*c*_L_ = *c*_M_ = 0.004 m). A constant pH of 7.7 (Cu^II^–H_2_dppa) or 7.3 (Cu^II^–H_4_dppp) was adjusted using 0.4 M morpholine/HCl buffer, 20 samples with changing concentration of 2,3,2-tet (0–0.02 M), at a final sample volume of 1 ml, equilibration time 1 h. Ionic strength was not strictly controlled. The protonation constants of 2,3,2-tet (log *K* = 10.08, 9.26, 6.88, 5.45) and the stability constant of its Cu^II^ complex (log *K* = 23.2) were taken from ref. 44. The stability constants of Cu^II^ complexes were determined by simultaneous treatment of the potentiometric and UV-vis competition data (absorbances at 535 nm and 840 nm as a function of 3,2,3-tet concentration) using the OPIUM program.^59^

### Single-crystal X-ray diffraction study

#### Preparation of single crystals of complexes with divalent metal ions

Hydrate of MCl_2_ (0.1 mmol) and ligand (0.1 mmol) were dissolved in water (2 ml). The pH of the solution was adjusted to 6–7 using 0.1 M aq. NaOH. All solutions were kept at the same pH, except the Cu^II^–H_4_dppp system, which was acidified to pH 4 using 0.1 M aq. HCl. The solutions were filtered through a microfilter into a 4 ml vial, and the filtrate was overlayered with an organic solvent (acetone, EtOH, *i*PrOH; 2 ml). Suitable crystals of [M(dpph)Cl_2_]·H_2_O·0.5*i*PrOH (M = Ni, Cu, Zn) were formed when *i*PrOH was used; in other cases, experiments with acetone were successful. The crystals were formed after two days.

#### Preparation of single crystals of complexes with trivalent metal ions

Hydrates of CoCl_2_ or FeCl_3_ or GaCl_3_ (0.1 mmol) and H_2_dppa (0.1 mmol) were dispersed in anhydrous EtOH (2 ml). The mixture was heated at 70 °C overnight in air. The solid was filtered on a fine glass frit, washed with anhydrous EtOH and dried under vacuum. The powder was then transferred into a 4 ml vial and dissolved in water (1 ml). The solution was overlayered with acetone (2 ml). The crystals were formed after three days.

#### Data collection and crystal structure refinement

Single-crystal X-ray diffraction data at 100 ((H_4_dppp)·3H_2_O, [Co(dppa)]Cl·4.5H_2_O, [Fe(dppa)Cl]·4H_2_O, {[Ga(dppa)]_4_}Cl_4_·17H_2_O) or 120 K (all other structures) using a Bruker D8 VENTURE Kappa Duo diffractometer equipped with a PHOTON100 detector and an IµS micro-focus sealed Mo Kα radiation source (*λ* = 0.71073 Å), in combination with a Cryostream cooling device (Oxford Cryosystems). Data integration and reduction were performed using the SAINT software package (Bruker AXS Inc.), followed by absorption correction employing a numerical method implemented in SADABS.^60^ Structural solutions were obtained *via* direct methods (SHELXT 2018),^61^ and all structures were refined by full-matrix least-squares techniques (SHELXL 2017).^62^

When possible, all non-hydrogen atoms were refined anisotropically (some atoms of the disordered parts with low occupancy were refined isotropically). All hydrogen atoms were found in the difference density map. However, the hydrogen atoms bound to the carbon atoms were fixed in theoretical positions using *U*_eq_(H) = 1.2 *U*_eq_(C) to keep the number of refined parameters low. Full refinement of the hydrogen atoms bound to oxygen or nitrogen atoms was attempted. Sometimes, these hydrogen atoms had to be fixed in the original position of electron maxima if their full refinement led to unrealistic bond lengths.

In the crystal structure of (H_4_dppp)·3H_2_O, one formula unit forms the structurally independent part. All hydrogen atoms bound to the nitrogen and oxygen atoms were fully refined. In two close water molecules of crystallisation, one hydrogen atom was split in two half-occupied positions, forming alternative hydrogen bonds between the corresponding oxygen atoms. In the isostructural series of [M(dpph)Cl_2_]·H_2_O·0.5*i*PrOH (M = Ni, Cu, Zn), one-half of the formula unit forms the independent part. The macrocyclic complex possesses two-fold symmetry. The oxygen atom of the water molecule of crystallisation lies in a special position with half-occupancy, and the molecule of *i*PrOH is disordered around a special position with quarter-occupancy. The hydrogen atom of the water molecule was fully refined, and that of *i*PrOH was fixed in the original position using *U*_eq_(H) = 1.2 *U*_eq_(O). In the crystal structure of [Ni(dppa)(H_2_O)_2_]·2H_2_O, one-half of the formula unit forms the independent part. The molecule has two-fold symmetry. In the isostructural phases [M(dppa)]·2H_2_O (M = Ni, Cu), one formula unit forms the structurally independent part. One of the water molecules of crystallisation in the structure of the Cu^II^ complex was refined disordered in two close positions; the hydrogen atoms of this molecule were fixed in the original positions using *U*_eq_(H) = 1.2 *U*_eq_(O). Other water hydrogen atoms were fully refined. In the crystal structure of dimeric {[Cu(H_2_dppp)]_2_}·10H_2_O·2CH_3_COCH_3_, one half of the formula unit forms the structurally independent part. All oxygen-bound hydrogen atoms were fully refined. However, the acetone molecule of crystallisation was found disordered; the disorder was refined by splitting the methyl carbon atoms into two positions (refined occupancies 51 : 49%) and fixing their hydrogen atoms using *U*_eq_(H) = 1.5 *U*_eq_(C), with the keto group shared. In the crystal structure of [Co(dppa)]Cl·4.5H_2_O, one formula unit forms the structurally independent part. The chloride counterion was found disordered. The disorder was treated by splitting into three close positions with refined occupancies 48 : 46 : 6%. The hydrogen atoms belonging to the water molecules were fixed in the original positions using *U*_eq_(H) = 1.2 *U*_eq_(O). In the crystal structure of [Fe(dppa)Cl]·4H_2_O, one formula unit forms the structurally independent part. A disorder of the water molecules of crystallisation was found, which could not be reliably modelled, and, thus, Platon SQUEEZE^63^ was used to subtract partial electron density corresponding to two water molecules from the independent part. The other two molecules were refined with hydrogen atoms fixed in the original positions (two hydrogen atoms split in two positions) with refined thermal factors. In the crystal structure of {[Ga(dppa)]_4_}Cl_4_·17H_2_O, one half of the tetramer forms the independent part. A complicated disorder was found in the channels formed in the crystal packing; the chloride counterions were split in several positions, and several water molecules of crystallisation were located, with their hydrogen atoms fixed in the original positions, using both thermal factor refinement and *U*_eq_(H) = 1.2 *U*_eq_(O) for some of them. Some remaining hard-to-interpret electron maxima of the disordered molecules were treated with Platon SQUEEZE;^63^ the subtracted density corresponds to 0.45Cl and 4.5H_2_O per the independent part.

## Conflicts of interest

There are no conflicts to declare.

## Supplementary Material

RA-016-D6RA00469E-s001

RA-016-D6RA00469E-s002

## Data Availability

CCDC 2516498–2516508 contain the supplementary crystallographic data for this paper.^64*a*–*k*^ The data supporting this article have been included as part of the supplementary information (SI). Supplementary information: synthesis and NMR spectra of the studied ligands, solid-state structures and crystallographic data, overall protonation and stability constants, NMR and UV-vis titrations and distribution diagrams. See DOI: https://doi.org/10.1039/d6ra00469e.
