# Supplementary material for: Dipyridinophane ligands – synthesis and coordination study
Source: RSC Adv. 2026 Apr 7;16(20):18220–31. doi: 10.1039/d6ra00469e (PMC13054815; doi:10.1039/d6ra00469e)
Supplement: RA-016-D6RA00469E-s001 [file RA-016-D6RA00469E-s001.pdf]

Supplementary Information (SI) for RSC Advances.
This journal is © The Royal Society of Chemistry 2026

Supplementary information

Dipyridinophane ligands – synthesis and coordination study

Lucie Kuncová, Jana Lazarová, Jan Kotek, Vojtěch Kubíček,* Petr Hermann

Department of Inorganic Chemistry, Faculty of Science, Charles University in Prague, Hlavova 2030, 128 40 Prague (Czech Republic), E-mail: kubicek@natur.cuni.cz

Table of contents

Synthesis of the studied ligands

2,6-bis(bromomethyl)pyridine (1)

2,6-bis(aminomethyl)pyridine (4)

2,6-bis{[(4-methylphenyl)sulfonamido]methyl}pyridine (2)

NMR spectra of the studied ligands

Figure S1. The ^{1}H and $^{13}C\{^{1}H\}$ NMR spectra of **dpph**.

Figure S2. The ^{1}H and $^{13}C\{^{1}H\}$ NMR spectra of H_2**dppa**.

Figure S3. The ^{1}H-^{13}C HSQC and HMBC NMR spectra of H_2**dppa**.

Figure S4. The ^{1}H and $^{13}C\{^{1}H\}$ NMR spectra of H_4**dppp**.

Figure S5. The ^{31}P NMR spectra of H_4**dppp**.

Figure S6. The ^{1}H-^{13}C HSQC and HMBC NMR spectra of H_4**dppp**.

Mass Spectra (ESI)

Figure S7. Mass spectrum of **compound 3**

Figure S8. Mass spectrum of **dpph**

Figure S9. Mass spectrum of **H_2dppa**

Figure S10. Mass spectrum of **H_4dppp**

Figure S11. Mass spectrum of **[Ni(dpp)Cl_2]**

Figure S12. Mass spectrum of **[Cu(dpp)Cl_2]**

Figure S13. Mass spectrum of **[Zn(dpp)Cl_2]**

Figure S14. Mass spectrum of **[Fe(dppa)Cl]**

Figure S15. Mass spectrum of **[Co(dppa)]Cl**

Figure S16. Mass spectrum of **[Ni(dppa)]** (same as for **[Ni(dppa)(H_2O)$_2$]**)

Figure S17. Mass spectrum of **[Cu(dppa)]**

Figure S18. Mass spectrum of **[Zn(dppa)]**

Figure S19. Mass spectrum of **{[Ga(dppa)]$_4$}Cl$_4$**

Figure S20. Mass spectrum of **[Ni(H_2dppp)]**

Figure S21. Mass spectrum of **{[Cu(H_2dppp)]**

Solid-state structures

Figure S22. Molecular structure of H_4**dppp** found in the crystal structure of H_4**dppp**·3H_2O.

Overall protonation constants of the studied ligands and stability constants of their complexes

Table S1. Overall protonation constants logβ of the studied ligands.

Table S2. Overall stability constants logβ of complexes with the studied.

NMR titrations of the Zn^{II}–ligand systems

Figure S23. The ^{1}H NMR titration of the Zn^{II}–H_2**dppa** system.

Figure S24. The ^{1}H NMR and $^{31}P\{^1H\}$ NMR titration of the Zn^{II}–H_4**dppp** system.

UV-VIS spectroscopic titration of the Cu^{II}–ligand systems

Figure S25. The competitive UV-VIS titration of the Cu^{II}–H_2**dppa–2,3,2-tet** system.

Figure S26. The competitive UV-VIS titration of the Cu^{II}–H_4**dppp–2,3,2-tet** system.

Figure S27. The distribution diagram of the ternary Cu^{II}–H_2**dppa–2,3,2-tet** system.

Distribution diagrams of M^{II}–ligand systems

Figure S28. Distribution diagram of the Ni^{II}–**dpph** system.

Figure S29. Distribution diagram of the Ni^{II}–H_2**dppa** system.

Figure S30. Distribution diagram of the Ni^{II}–H_4**dppp** system.

Figure S31. Distribution diagram of the Zn^{II}–**dpph** system.

Figure S32. Distribution diagram of the Zn^{II}–**H_2dppa** system.

Figure S33. Distribution diagram of the Zn^{II}–**H_4dppp** system.

Crystallography

Table S3. Crystallographic parameters of the studied compounds.

Table S4. Bond lengths and angles of the metal coordination sphere in the solid state.

Synthesis of the studied ligands

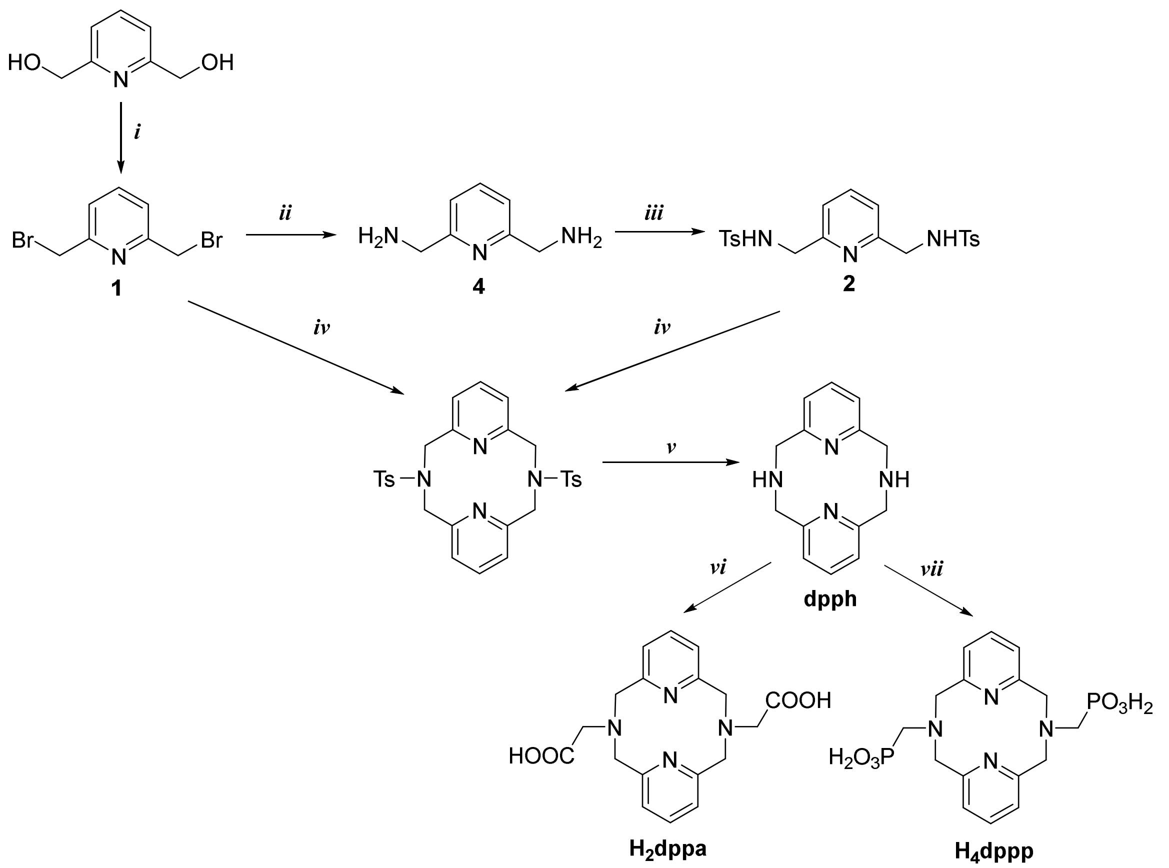

Scheme S1. Synthesis of ligands: (*i*): 33% HBr in AcOH, 100 °C. (*ii*): 1. potassium phthalimide, DMF, 100 °C, 2 d; 2. $N_2H_4 \cdot H_2O$, EtOH, reflux, 1 d. (*iii*): TsCl, dichloromethane, Et_3N, 1 d. (*iv*): tetrabutylammonium iodide, $LiOH \cdot H_2O$, dichloromethane/H_2O, 45 °C, 12 h. (*v*): 98% H_2SO_4, 110 °C, 4 h. (*vi*): chloroacetic acid, $LiOH \cdot H_2O$, H_2O, 60 °C, 1 d. (*vii*): 1. diethylphosphite, paraformaldehyde, pyridinium hydrobromide, pyridine, 40 °C, 1 d; 2. 6 M aq. HCl, 80 °C, 4 d.

2,6-bis(bromomethyl)pyridine (1) [1]

A solution of 2,6-pyridinedimethanol (8.0 g, 57.5 mmol) in 33% HBr in glacial AcOH (100 ml) was heated at 100 °C under a condenser for 90 min. Upon completion, the reaction mixture was poured onto crushed ice (200 ml), forming a precipitate. The pH of the resulting suspension was carefully adjusted to 9 using a 1M aq. NaOH. The precipitated product was isolated by vacuum filtration, washed with cold water, and dried under reduced pressure to yield a white solid (12.4 g, 81%).

^{1}H NMR (DMSO-d$_6$): 7.83 (t, $^3J_{HH}$ 7.7, 1H, py), 7.48 (d, $^3J_{HH}$ 7.7, 2H, py), 4.67 (s, 4H, CH_2). ^{13}C{^{1}H} NMR (DMSO-d$_6$): 156.6 (s, py), 138.5 (s, py), 123.2 (s, py), 34.4 (s, CH_2). MS(+): *m*/*z* 265.9 $[M+H]^+$

2,6-bis(aminomethyl)pyridine (4) [2]

A stirred solution of **1** (10.0 g, 38 mmol) and potassium phthalimide (14.0 g, 75 mmol) in *N*,*N*-dimethylformamide (50 ml) was heated at 100 °C for 48 h. Upon completion, the reaction mixture was cooled to room temperature and diluted with deionised water (80 ml), forming a white precipitate. The solid was collected by vacuum filtration and re-suspended in EtOH (100 ml). Hydrazine hydrate (7.8 ml) was added, and the mixture was refluxed for 24 h. After cooling to room temperature, 6M aq. HCl (80 ml) was added, and the mixture was refluxed for 2 h, and then was stirred at room temperature for the next 10 h. The solids were filtered off, and the filtrate was evaporated under reduced pressure. The resulting residue was dissolved in distilled water (100 ml), and the pH of the solution was adjusted to 12 using 6M KOH. The solution was extracted with chloroform (5×100 ml). The combined organic layers were dried with Na_2SO_4, filtered and concentrated under reduced pressure. The product, obtained as a brown-green oil, crystallised upon standing (3.91 g, 75%).

^{1}H NMR ($CDCl_3$): 7.58 (t, $^3J_{HH}$ 7.6, 1H, py), 7.11 (d, $^3J_{HH}$ 7.6, 2H, py), 3.93 (s, 4H, CH_2). ^{13}C{^{1}H} NMR ($CDCl_3$): 161.3 (s, py), 137.0 (s, py), 119.2 (s, py), 47.6 (s, CH_2). MS(+): *m/z* 138.1 $[M+H]^+$.

2,6-bis{[(4-methylphenyl)sulfonamido]methyl}pyridine (2) [3]

Triethylamine (6.4 ml, 45.6 mmol) and **4** (3.0 g, 21.7 mmol) were dissolved in dichloromethane (100 ml), and the solution was cooled to 0 °C in an ice bath. A solution of *p*-toluenesulfonyl chloride (8.3 g, 45.6 mmol, 2.1 equiv.) in dichloromethane (100 ml) was added dropwise. After the addition was complete, the reaction mixture was allowed to warm to room temperature and stirred for 24 h. The organic phase was washed successively with distilled water (150 ml) and saturated aq. NaCl (100 ml), and dried with anhydrous $MgSO_4$. After filtration, the solvent was removed under reduced pressure to yield a crude orange solid. The crude product was purified by column chromatography on silica gel using a mixture of dichloromethane and ethyl acetate (9:1, v/v) as the eluent. Evaporation of solvent gave the product as a light yellow powder (8.91 g, 92%).

^{1}H NMR ($CDCl_3$): 7.69 (d, $^3J_{HH}$ 7.2, 4H, Ts), 7.48 (t, $^3J_{HH}$ 7.7, 1H, py), 7.19 (d, $^3J_{HH}$ 7.2, 4H, Ts), 7.02 (d, $^3J_{HH}$ 7.7, 2H, py), 6.02 (t, $^3J_{HH}$ 5.8, 2H, NH), 4.14 (d, $^3J_{HH}$ 5.8, 4H, CH_2), 2.36 (s, 6H, CH_3). ^{13}C{^{1}H} NMR ($CDCl_3$): 154.8 (s, py), 143.5 (s,Ts), 137.7 (s,Ts), 136.8 (s, py), 129.7 (s, Ts), 127.3 (s, Ts), 120.8 (s, py), 47.4 (s, CH_2), 21.6 (s, CH_3). MS(+): *m/z* 446.1 $[M+H]^+$.

NMR spectra of the studied ligands

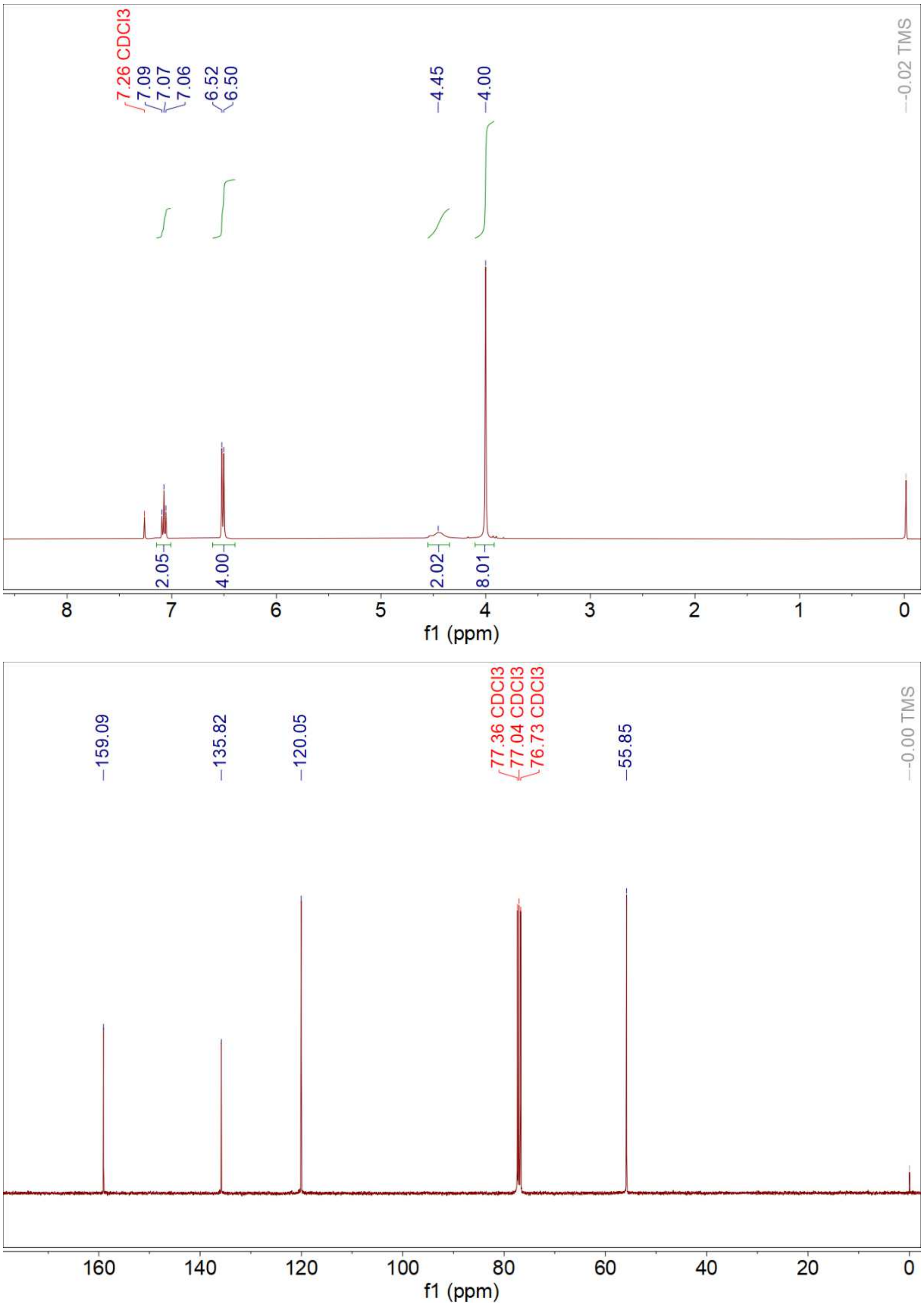

Figure S1. The ^{1}H (top) and $^{13}C\{^{1}H\}$ (bottom) NMR spectra of **dpph** in $CDCl_3$ (300 MHz, 25 °C).

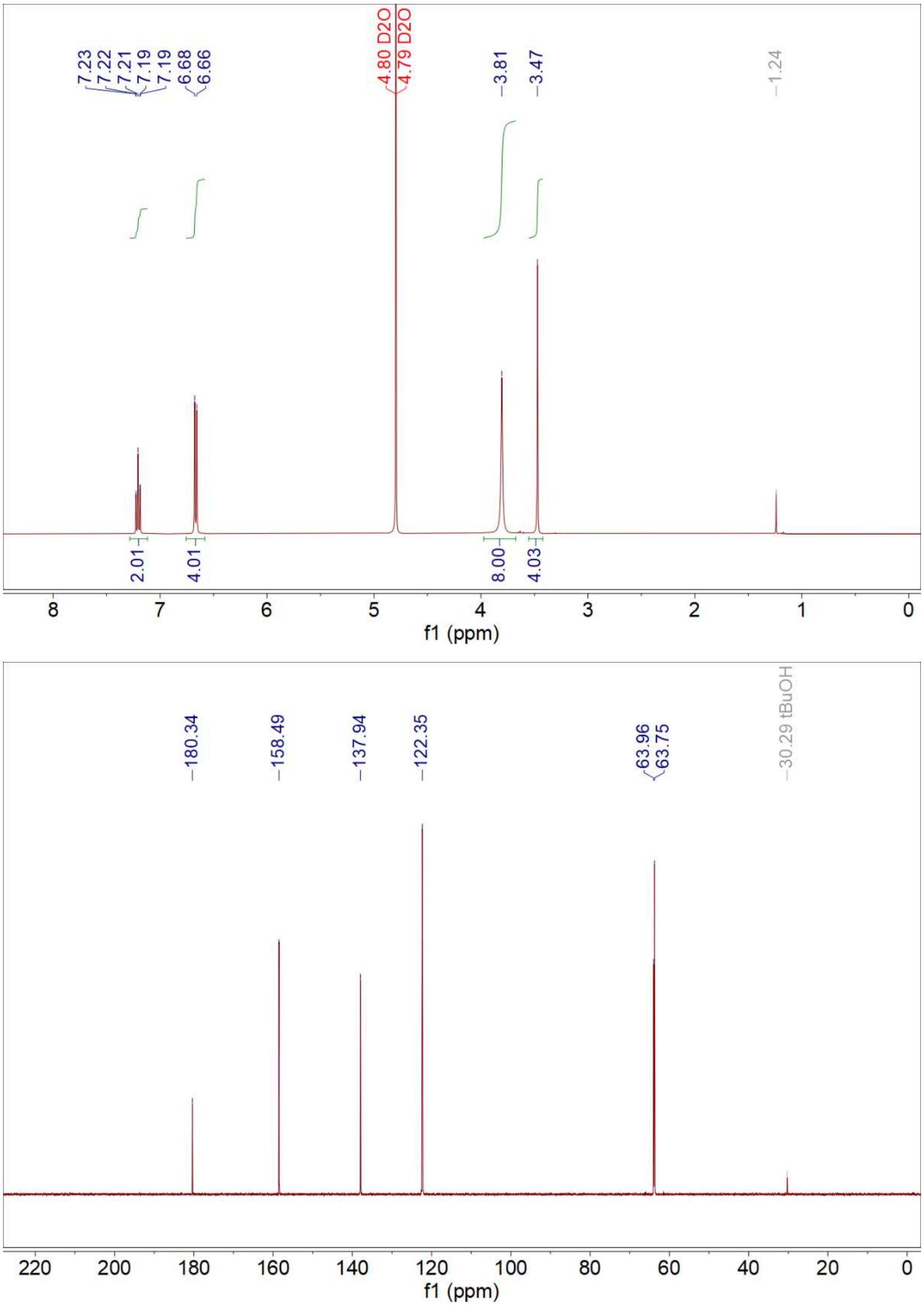

Figure S2. The ^{1}H (top) and ^{13}C{^{1}H} (bottom) NMR spectra of H_2**dppa** in D_2O/NaOH, pD ~13.0 (300 MHz, 25 °C).

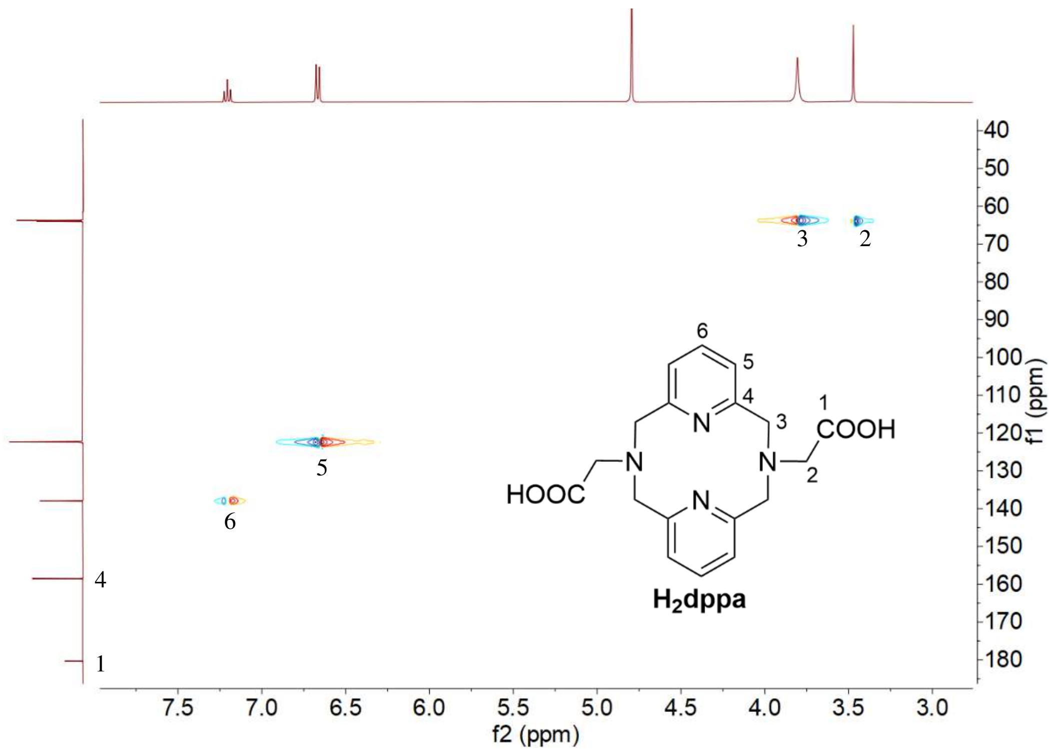

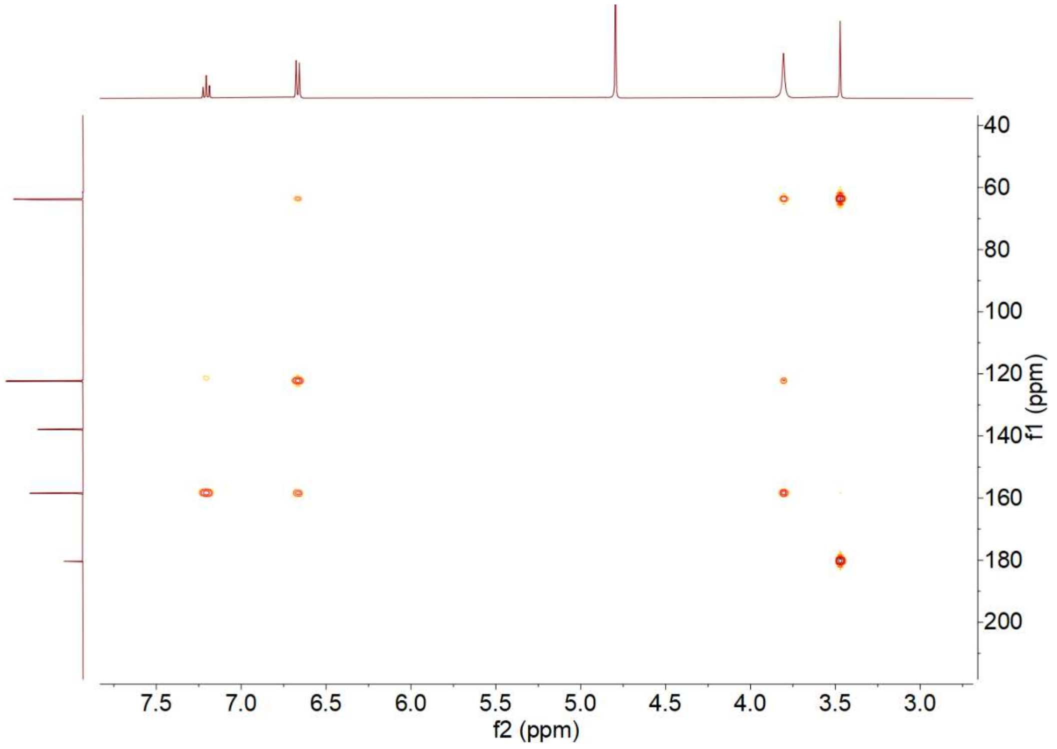

Figure S3. The ^{1}H-^{13}C HSQC (top) and HMBC (bottom) NMR spectra of H_2**dppa** in D_2O/NaOH, pD ~13.0 (300 MHz, 25 °C).

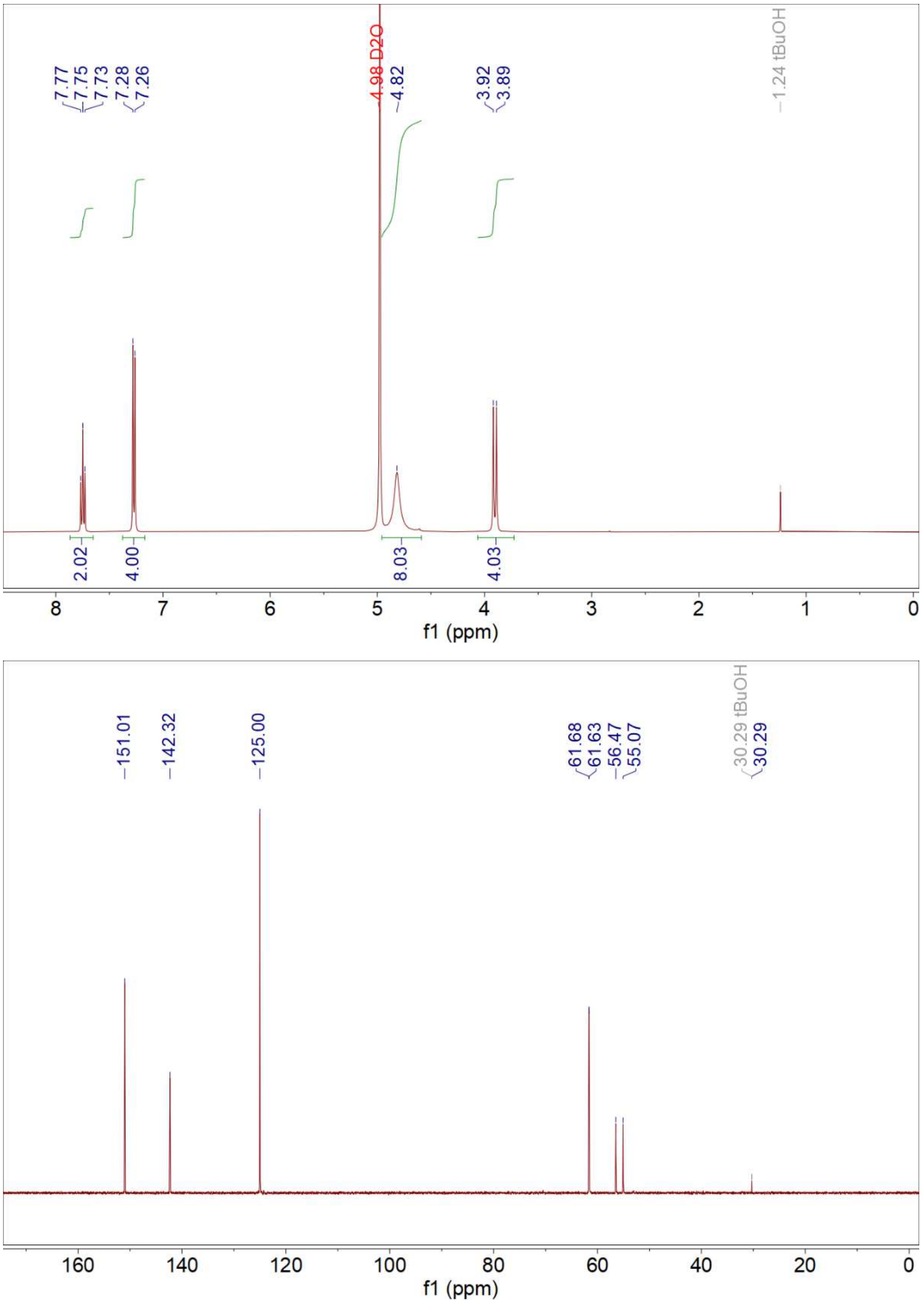

Figure S4. The ^{1}H (top) and ^{13}C{^{1}H} (bottom) NMR spectra of H_4**dppp** in D_2O/DCl, pH ~0.8 (300 MHz, 25 °C).

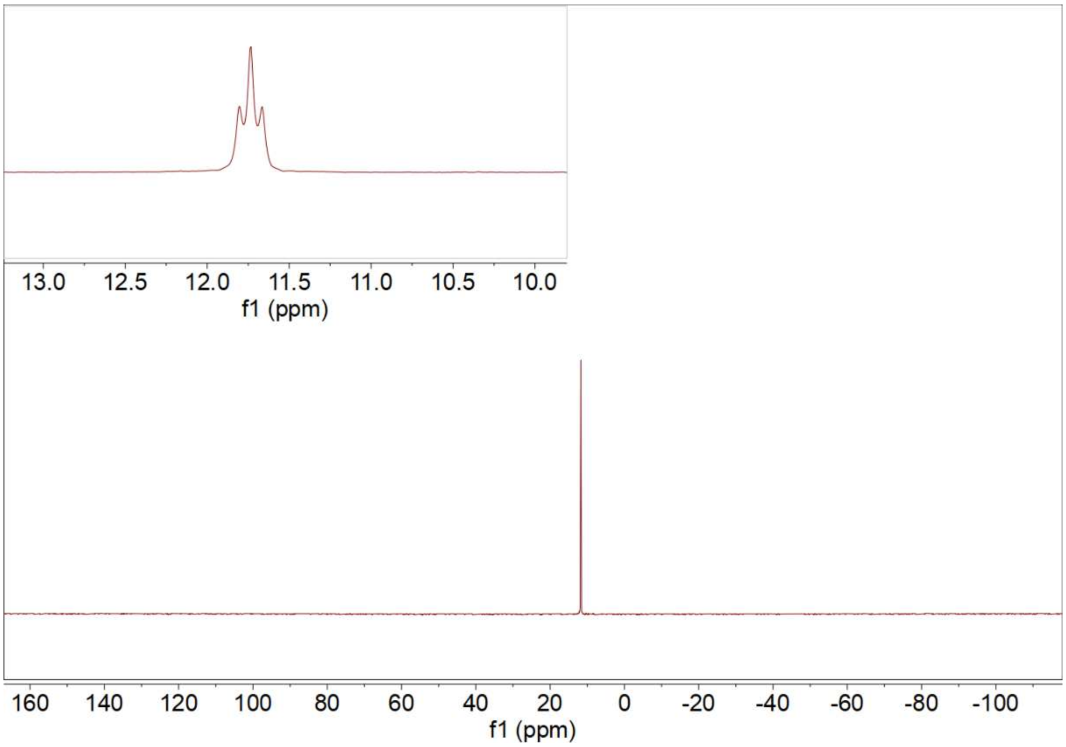

Figure S5. The $^{31}P\{^{1}H\}$ and ^{31}P (inset) NMR spectra of H_4**dppp** in D_2O/DCl, pH ~0.8 (300 MHz, 25 °C).

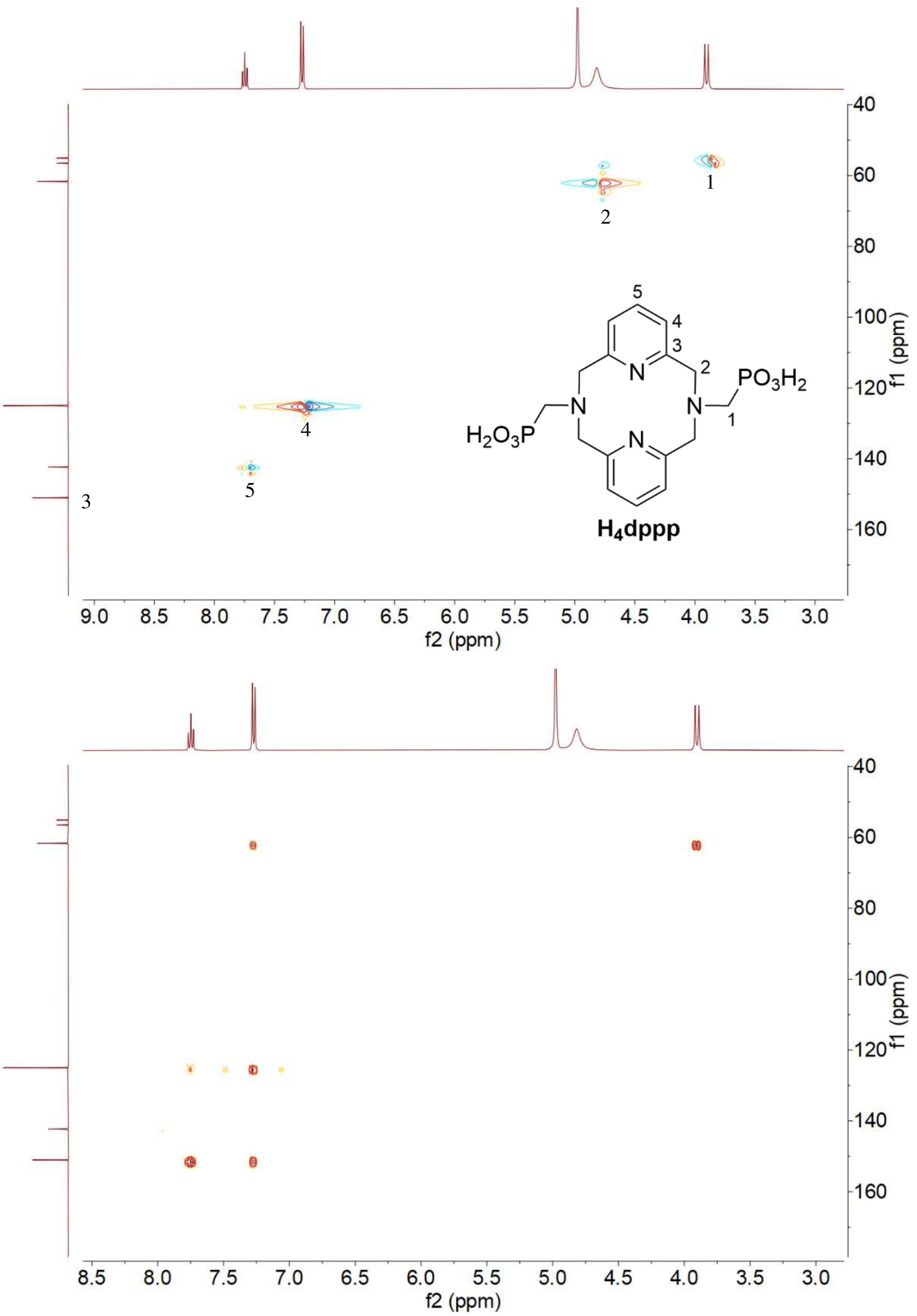

Figure S6. The ^{1}H-^{13}C HSQC (top) and HMBC (bottom) NMR spectra of H_4**dppp** in D_2O/DCl, pH ~0.8 (300 MHz, 25 °C).

Mass Spectra (ESI, positive mode)

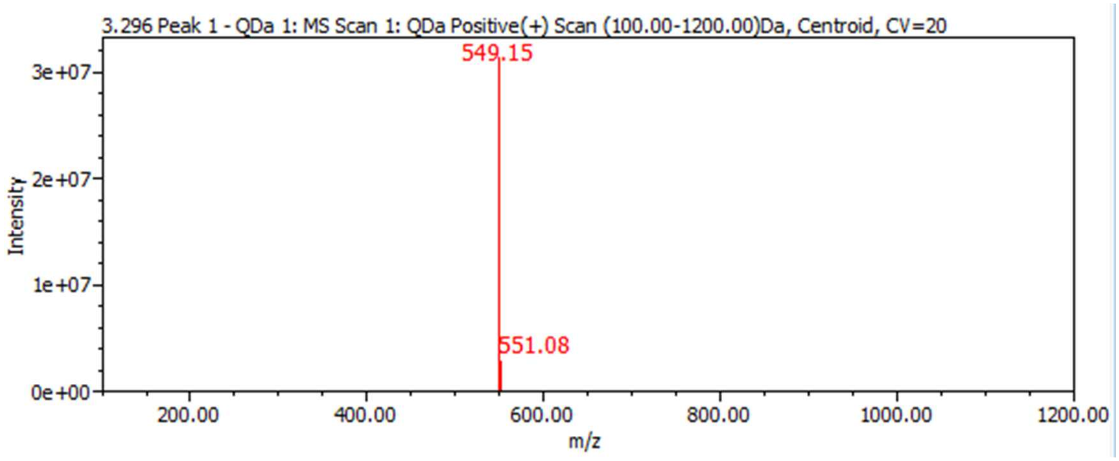

Figure S7. Mass spectrum of **compound 3**

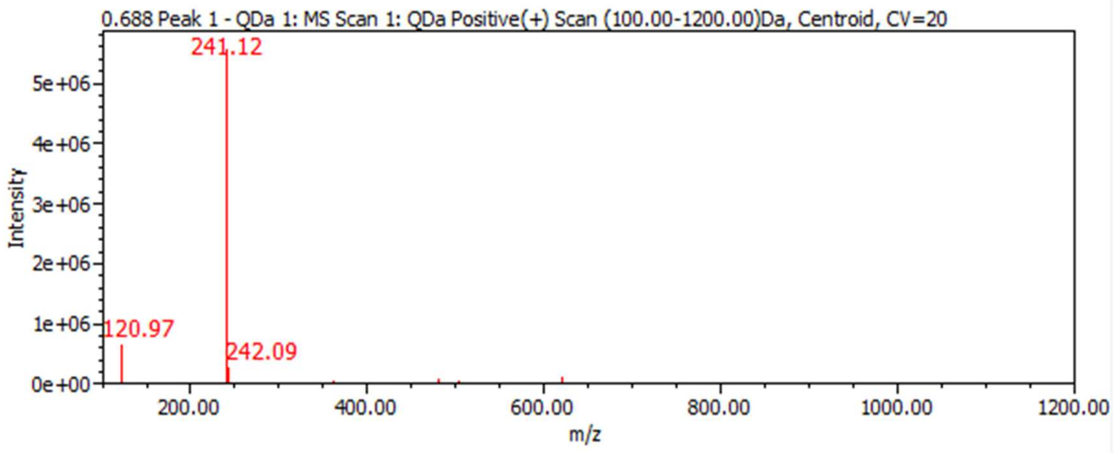

Figure S8. Mass spectrum of **dpph**

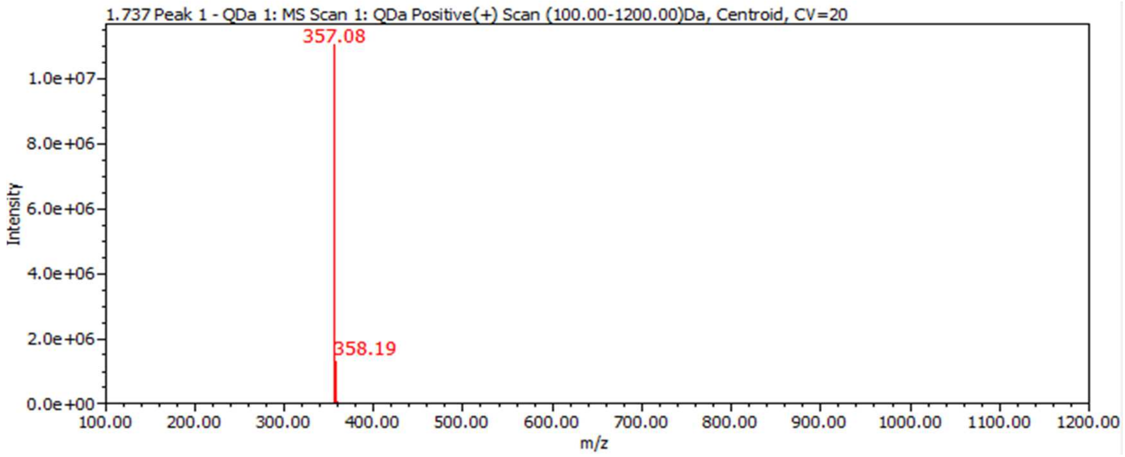

Figure S9. Mass spectrum of **H_2dppa**

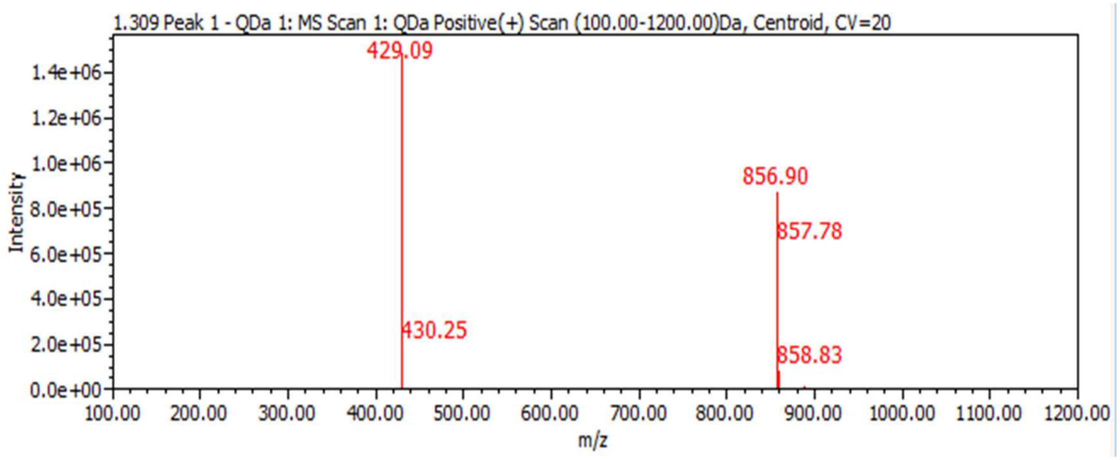

Figure S10. Mass spectrum of **H4dppp**

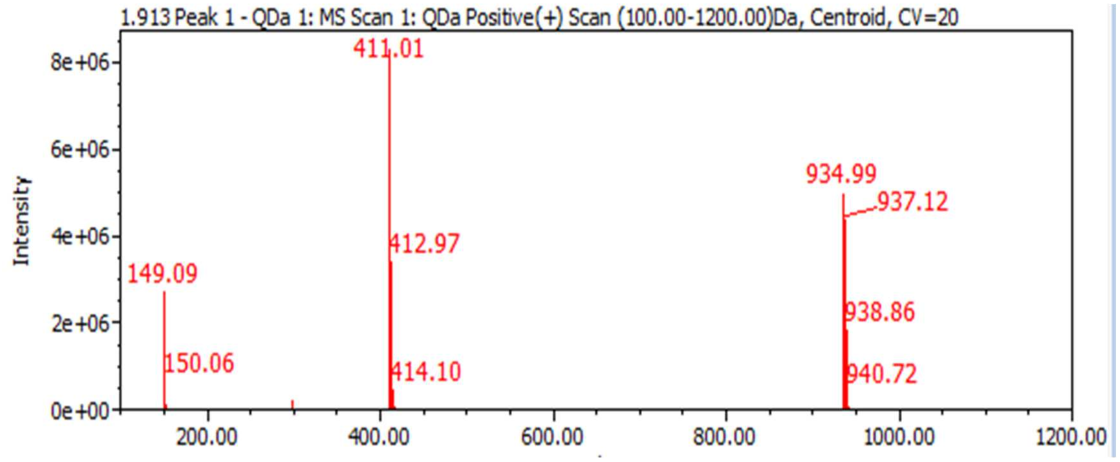

Figure S11. Mass spectrum of **[Ni(dpp)Cl$_2$]**

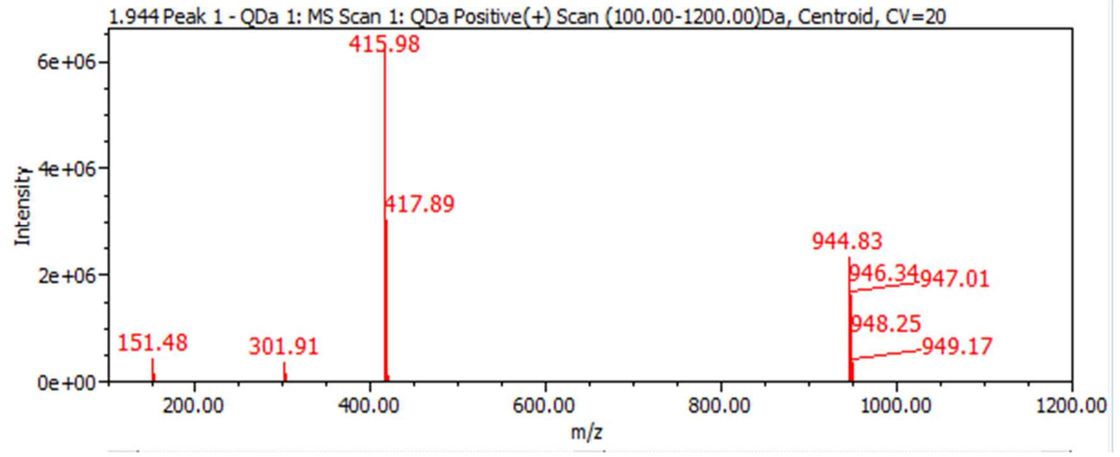

Figure S12. Mass spectrum of **[Cu(dpp)Cl$_2$]**

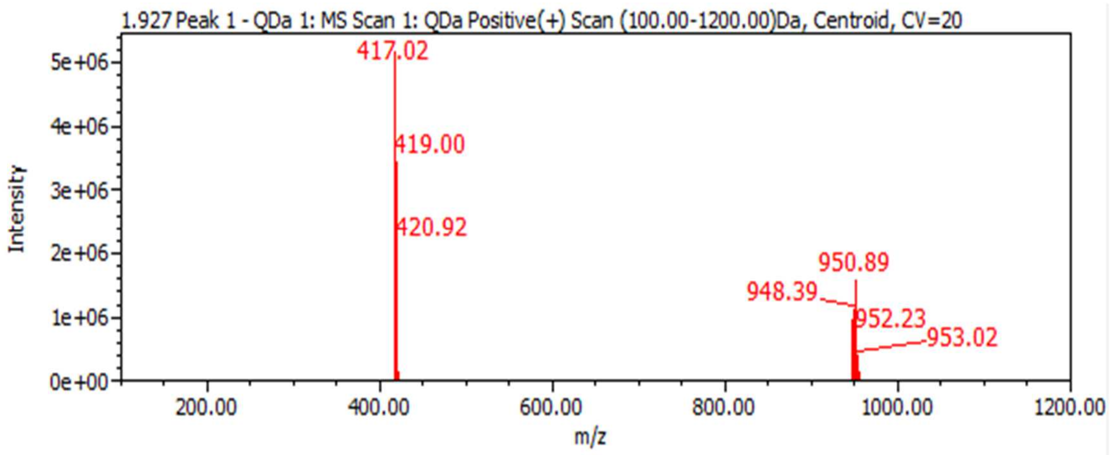

Figure S13. Mass spectrum of **[Zn(dpp)Cl$_2$]**

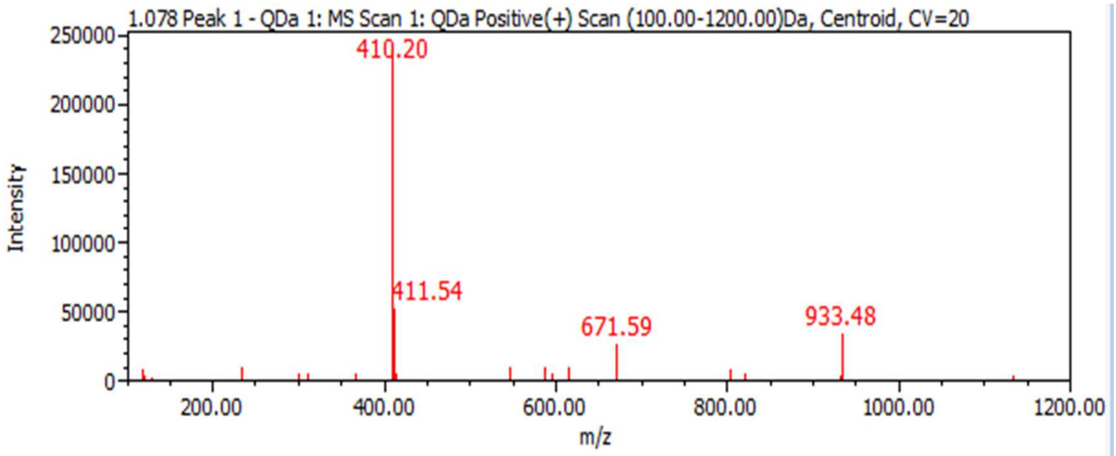

Figure S14. Mass spectrum of **[Fe(dppa)Cl]**

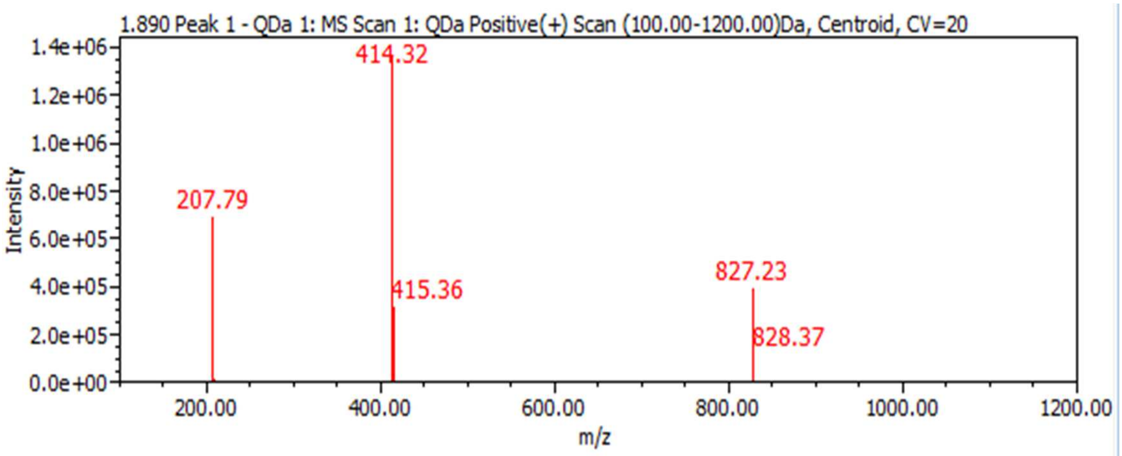

Figure S15. Mass spectrum of **[Co(dppa)]Cl**

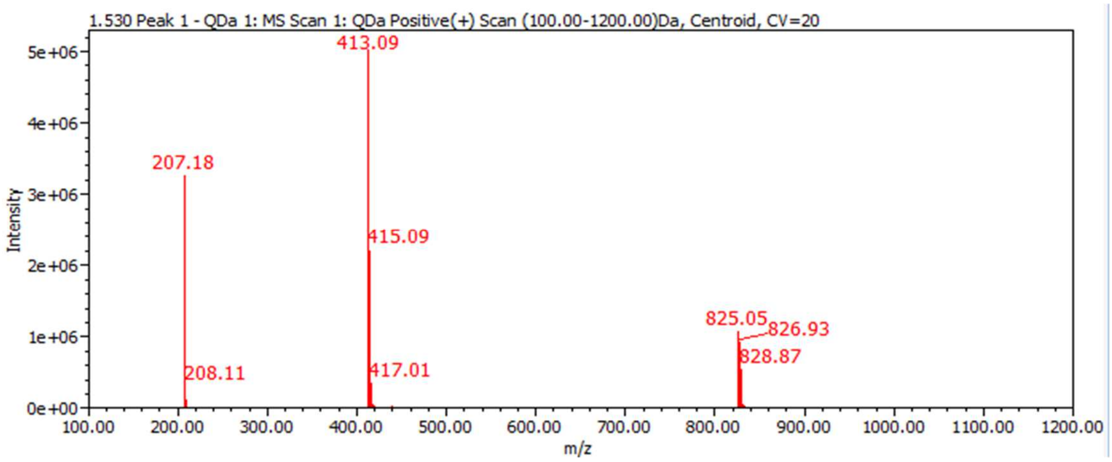

Figure S16. Mass spectrum of **[Ni(dppa)]** (same as for **[Ni(dppa)(H_2O)$_2$]**)

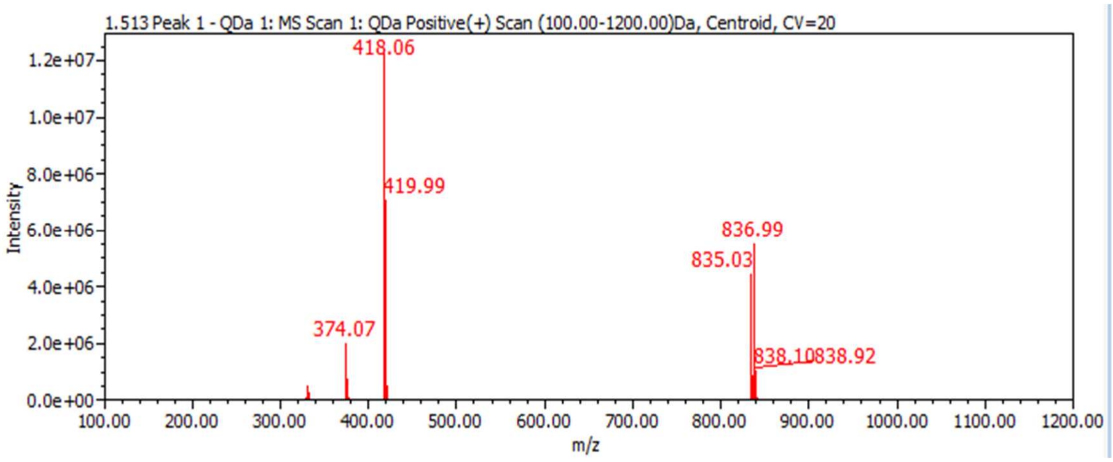

Figure S17. Mass spectrum of **[Cu(dppa)]**

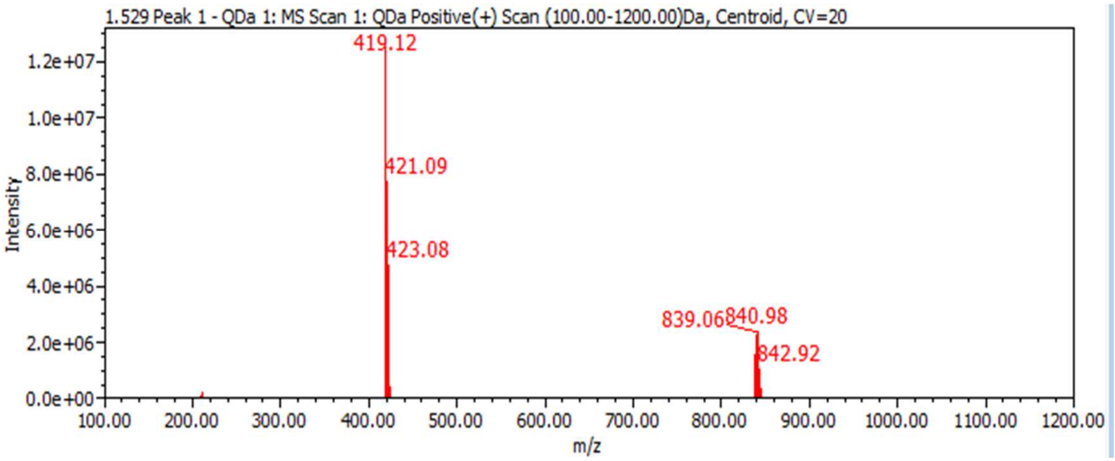

Figure S18. Mass spectrum of **[Zn(dppa)]**

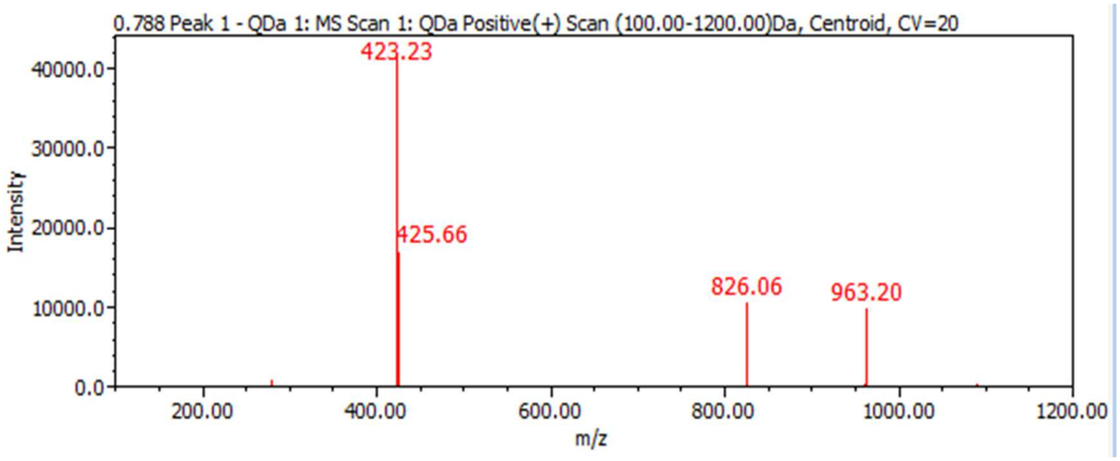

Figure S19. Mass spectrum of **{[Ga(dppa)]$_4$}Cl$_4$**

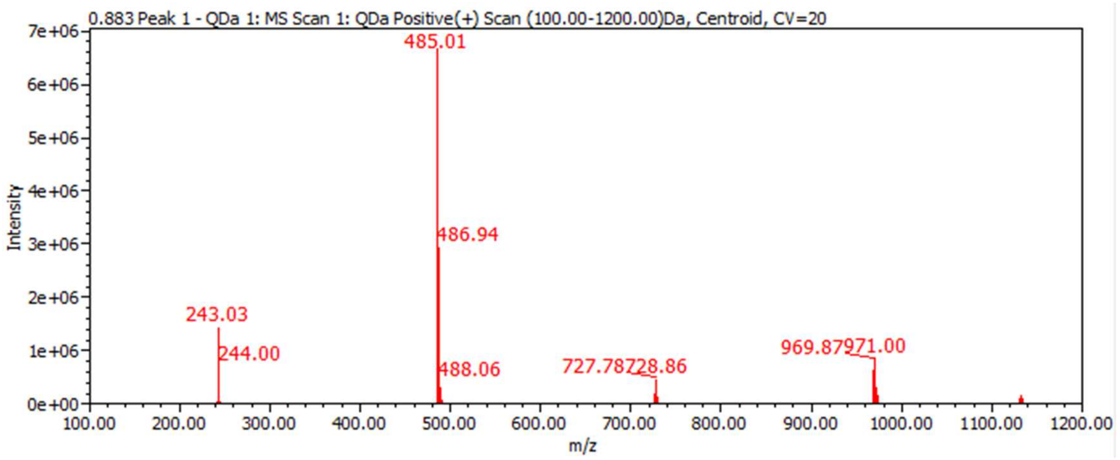

Figure S20. Mass spectrum of **[Ni(H$_2$dppp)]**

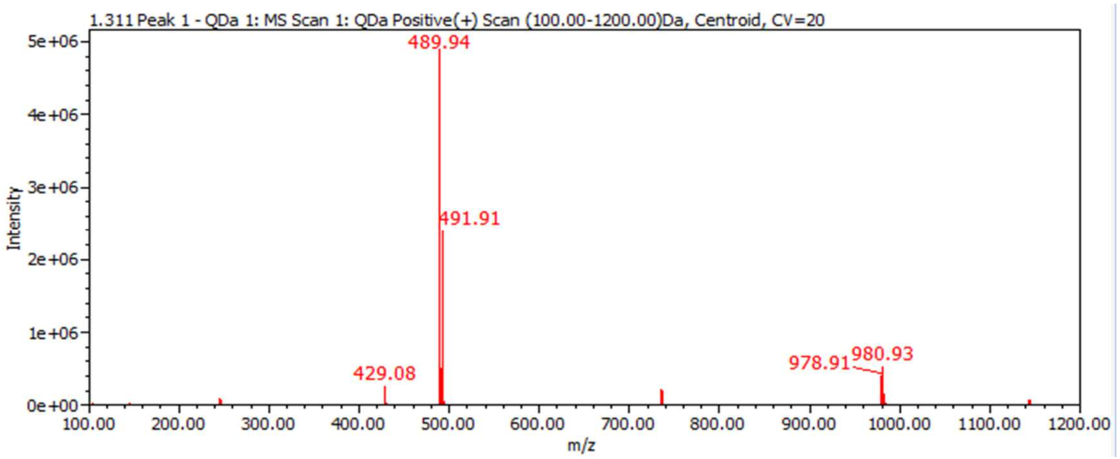

Figure S21. Mass spectrum of **{[Cu(H$_2$dppp)]$_2$}**

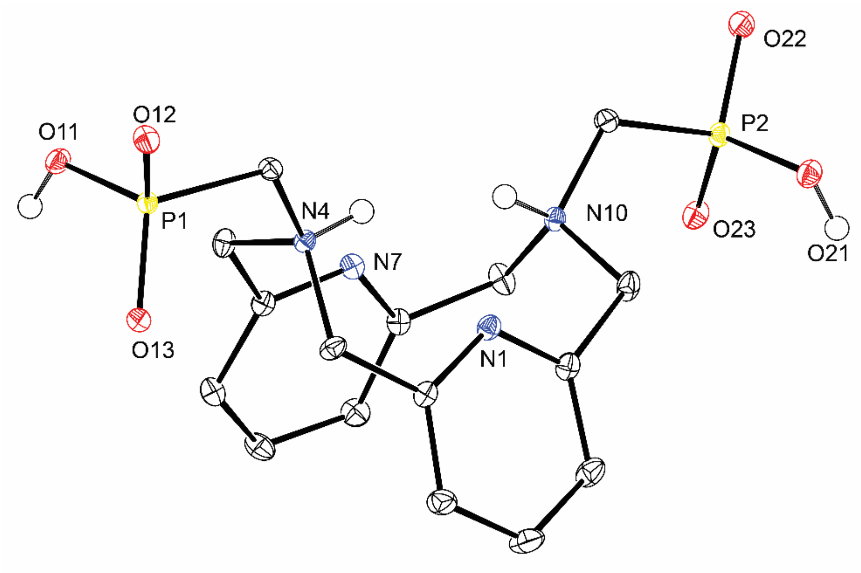

Figure S22. Molecular structure of H_4**dppp** found in the crystal structure of H_4**dppp**·$3H_2O$. The carbon-bound hydrogen atoms are not shown for clarity. Thermal ellipsoids are drawn at 50%.

Overall protonation constants of the studied ligands and stability constants of their complexes

Table S1. Overall protonation constants logβ of the studied ligands (25 °C, I = 0.1 M NMe_4Cl).

Species	**dpph**	H_2**dppa**	H_4**dppp**
HL	8.04(1)	9.62(1)	9.98(1)
H_2L	15.32(1)	15.51(1)	18.52(1)
H_3L	–	17.78(1)	24.96(1)
H_4L	–	19.11(2)	28.86(1)
H_5L	–	–	29.77(2)

Table S2. Overall stability constants logβ of complexes with the studied ligands (25 °C, I = 0.1 M NMe_4Cl).

		Metal ion		
Ligand	Equilibrium	Ni^{II}	Cu^{II}	Zn^{II}
**dpph**	M + L ⇄ [M(L)]	14.54(2)	15.98(2)	13.09(1)
	M + L + H_2O ⇄ [M(L)(OH)] + H^+	3.18(2)	7.10(2)	3.49(1)
	M + L + 2H_2O ⇄ [M(L)$(OH)_2$] + 2H^+	–	–5.47(2)	–
H_2**dppa**	M + L ⇄ [M(L)]	14.29(3)	20.48(1)	17.50(3)
	M + L + H^+ ⇄ [M(HL)]	17.12(3)	22.72(2)	19.85(2)
	M + L + 2H^+ ⇄ [M(H_2L)]	–	24.76(2)	–
	M + L + H_2O ⇄ [M(L)(OH)] + H^+	–	9.16(2)	5.20(3)
H_4**dppp**	M + L ⇄ [M(L)]	17.51(5)	22.64(2)	18.93(6)
	M + L + H^+ ⇄ [M(HL)]	24.05(4)	28.84(2)	24.73(5)
	M + L + 2H^+ ⇄ [M(H_2L)]	29.01(4)	33.60(3)	29.18(3)
	M + L + 3H^+ ⇄ [M(H_3L)]	–	34.97(3)	

NMR titrations of the Zn^{II}–ligand systems

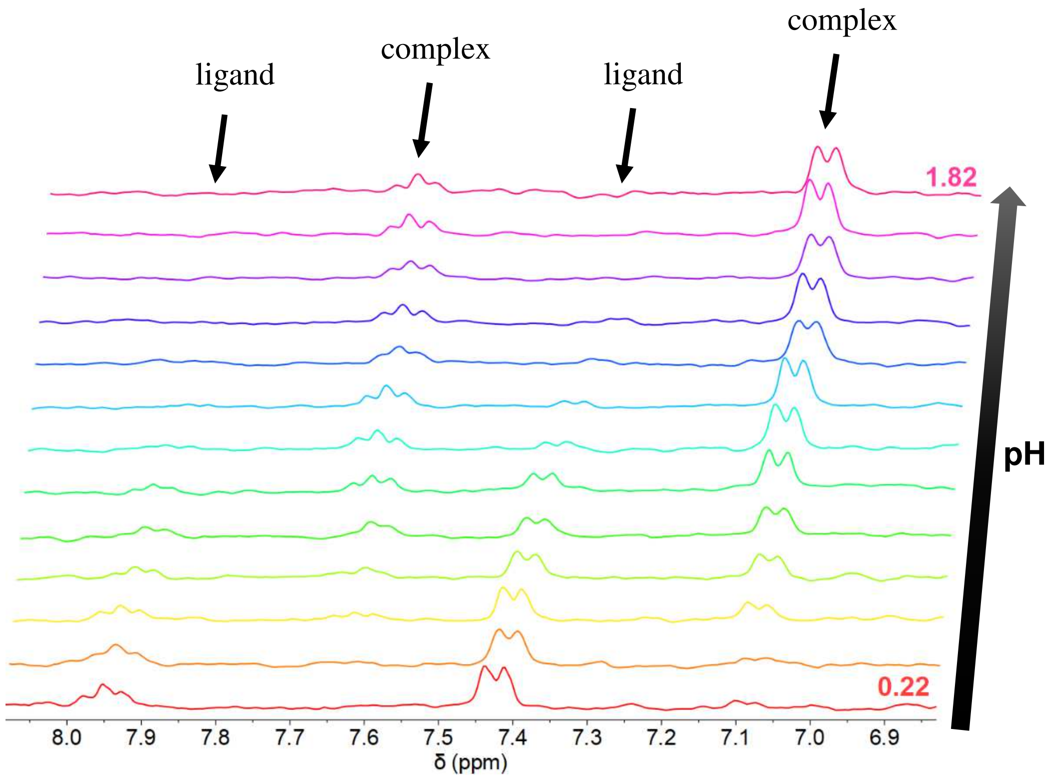

Figure S23. The ^{1}H NMR titration of the Zn^{II}–H_2**dppa** system ($c_M = c_L$ = 4 mM, pH 0.22–1.82, 300 MHz, 25 °C).

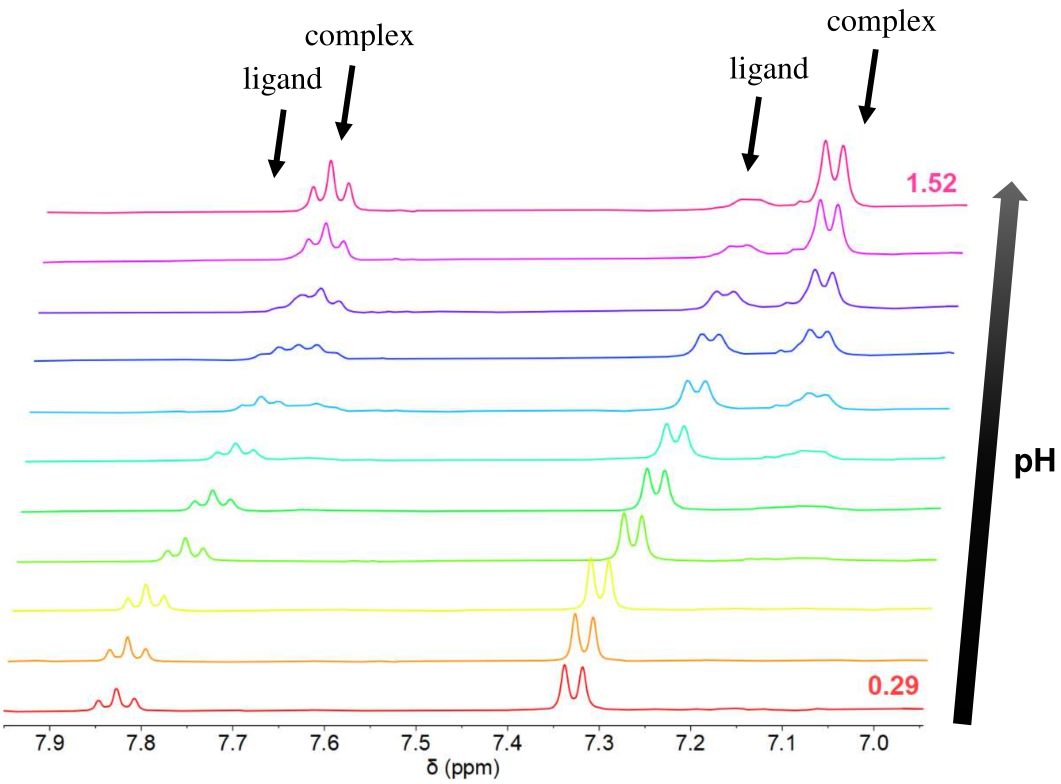

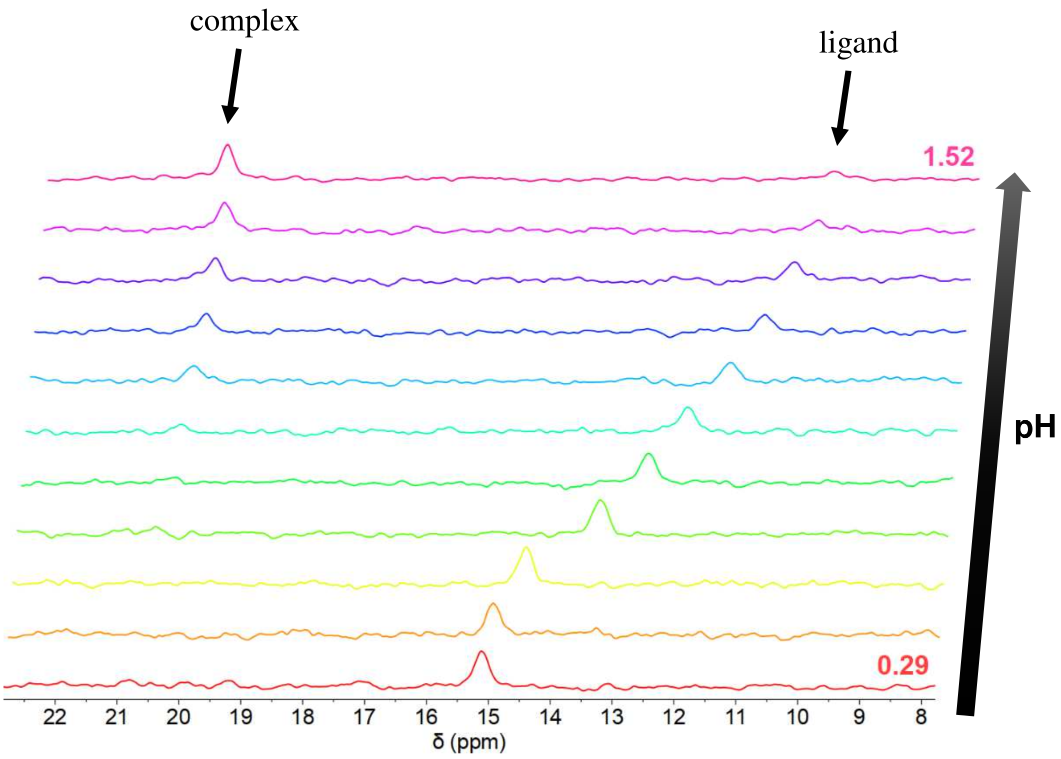

Figure S24. The ^{1}H NMR (top) and $^{31}P\{^{1}H\}$ NMR (bottom) titration of the Zn^{II}–H_4**dppp** system ($c_M = c_L$ = 4 mM, pH 0.29–1.52, 25 °C).

UV-VIS spectroscopic titration of the Cu^{II}–ligand systems

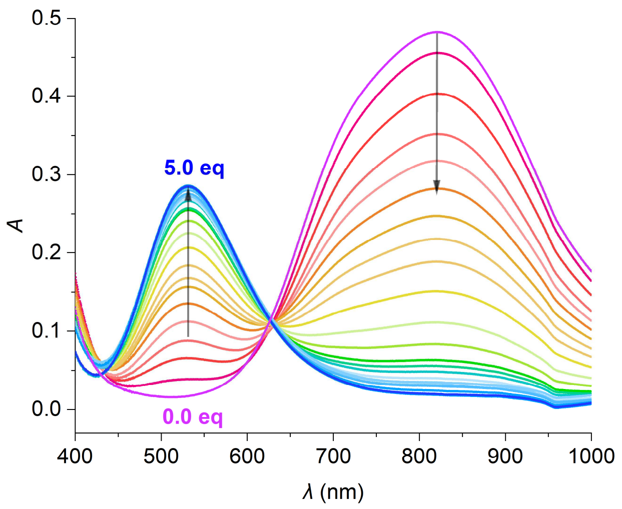

Figure S25. The competitive UV-VIS titration of the Cu^{II}–H_2**dppa–2,3,2-tet** system (c_{Cu} = 4 mM, $c_{\mathbf{dppa}}$ = 4 mM, $c_{\mathbf{232\text{-}tet}}$ = 0–20 mM, pH 7.7, 25 °C).

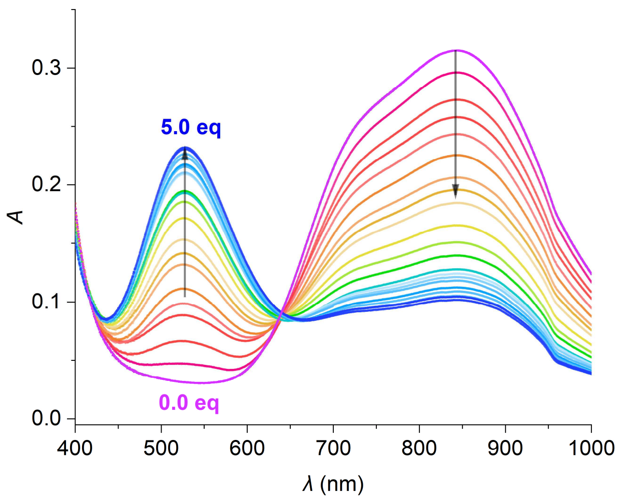

Figure S26. The competitive UV-VIS titration of the Cu^{II}–H_4**dppp–2,3,2-tet** system (c_{Cu} = 4 mM, $c_{\mathbf{dppp}}$ = 4 mM, $c_{\mathbf{232\text{-}tet}}$ = 0–20 mM, pH 7.3, 25 °C).

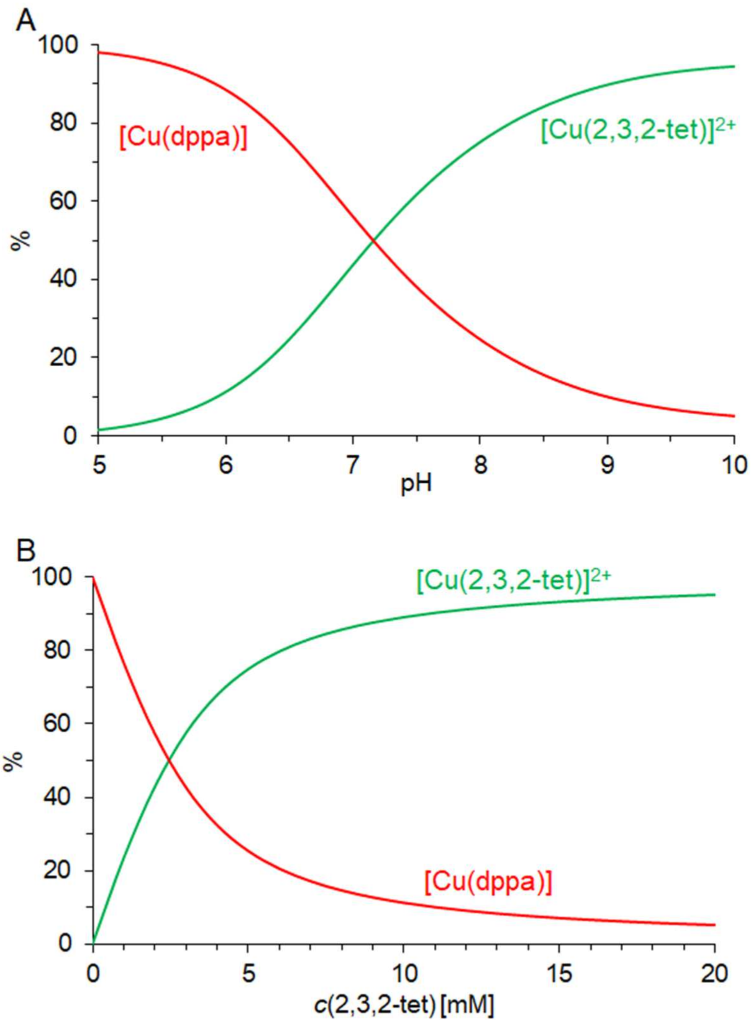

Figure S27. The distribution diagram of the ternary Cu^{II}–H_2**dppa–2,3,2-tet** system as function in the neutral region of pH (**A**, c_{Cu} = 4 mM, $c_{\mathbf{dppa}}$ = 4 mM, $c_{\mathbf{232\text{-}tet}}$ = 4 mM) or as function of **2,3,2-tet** concentration (**B**, c_{Cu} = 4 mM, $c_{\mathbf{dppa}}$ = 4 mM, pH 7.7).

Distribution diagrams of M^{II}–ligand systems

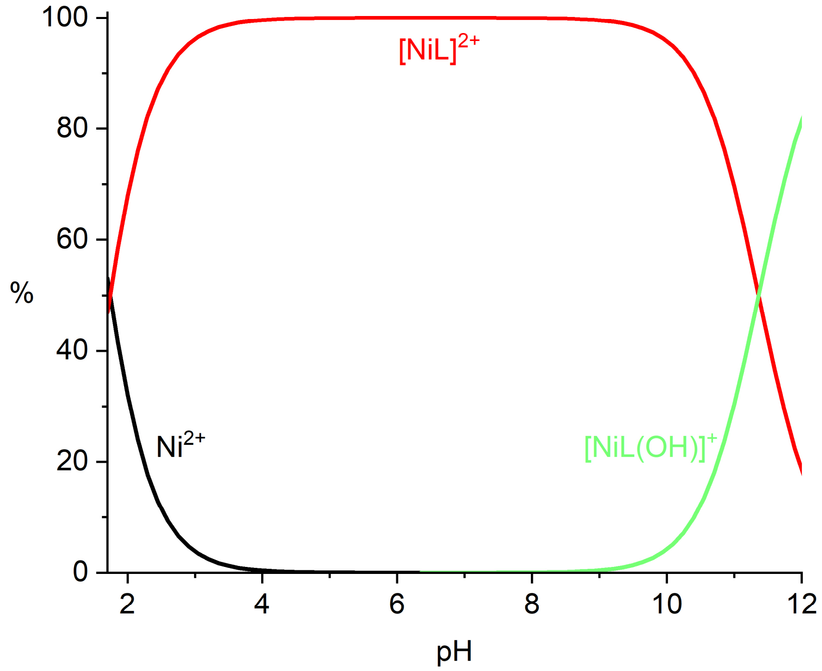

Figure S28. Distribution diagram of the Ni^{II}–**dpph** system ($c_M = c_L = 4$ mM, 25 °C).

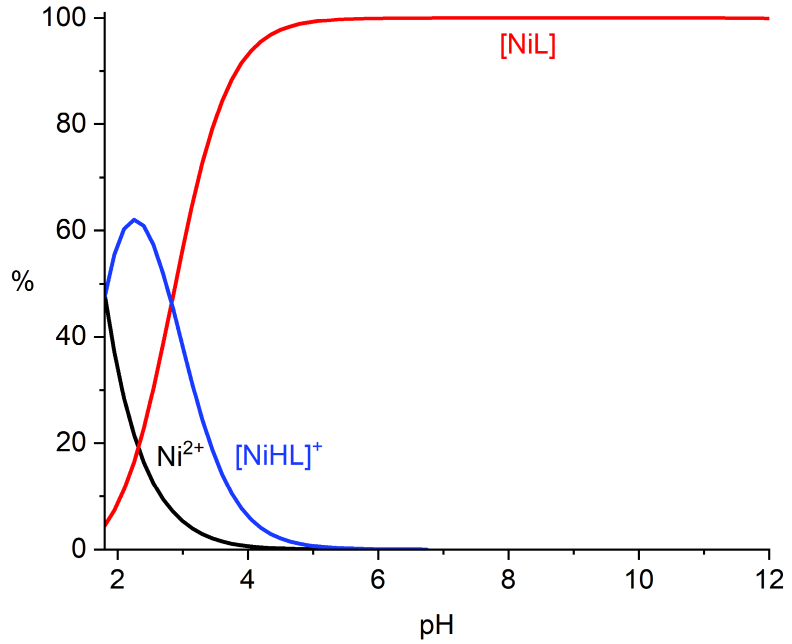

Figure S29. Distribution diagram of the Ni^{II}–H_2**dppa** system ($c_M = c_L = 4$ mM, 25 °C).

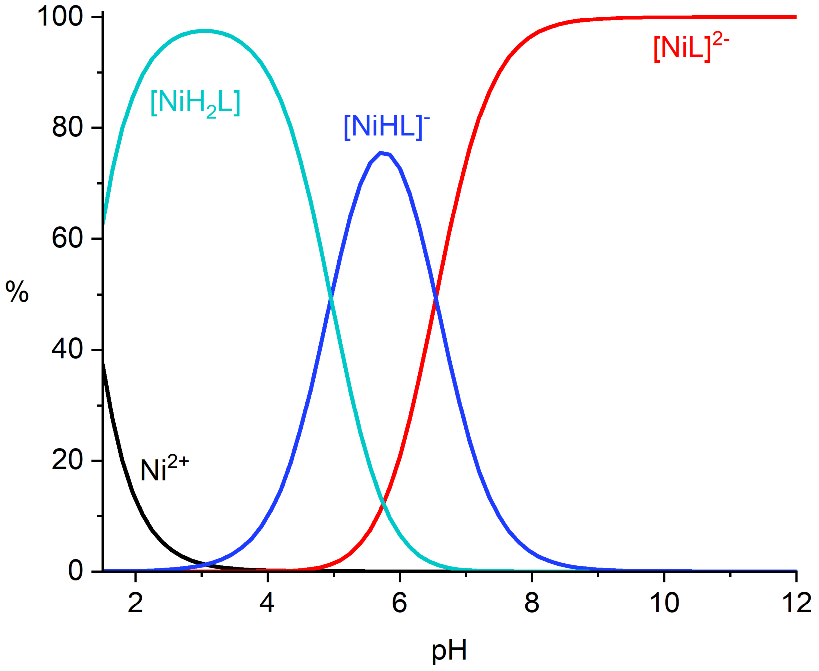

Figure S30. Distribution diagram of the Ni^{II}–H_4**dppp** system ($c_M = c_L$ = 4 mM, 25 °C).

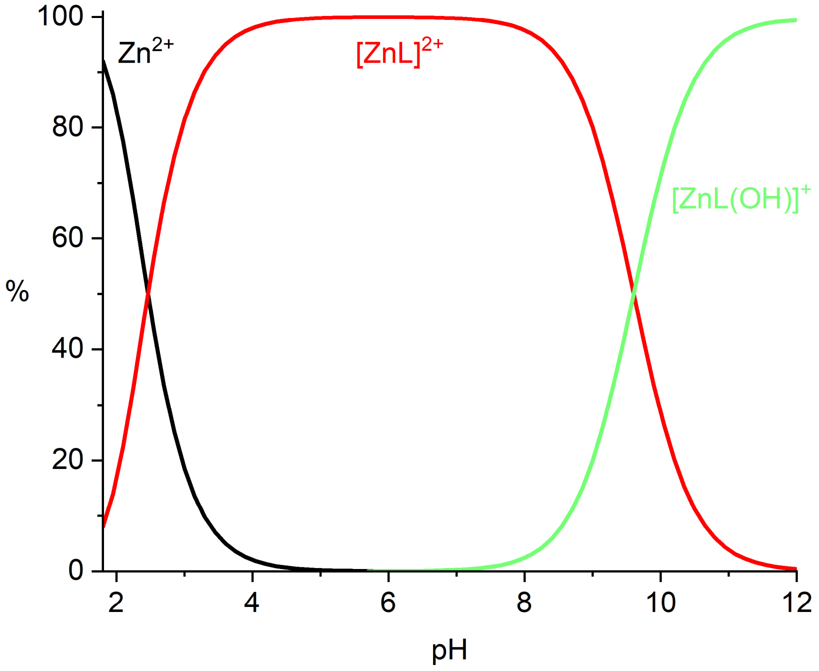

Figure S31. Distribution diagram of the Zn^{II}–**dpph** system ($c_M = c_L$ = 4 mM, 25 °C).

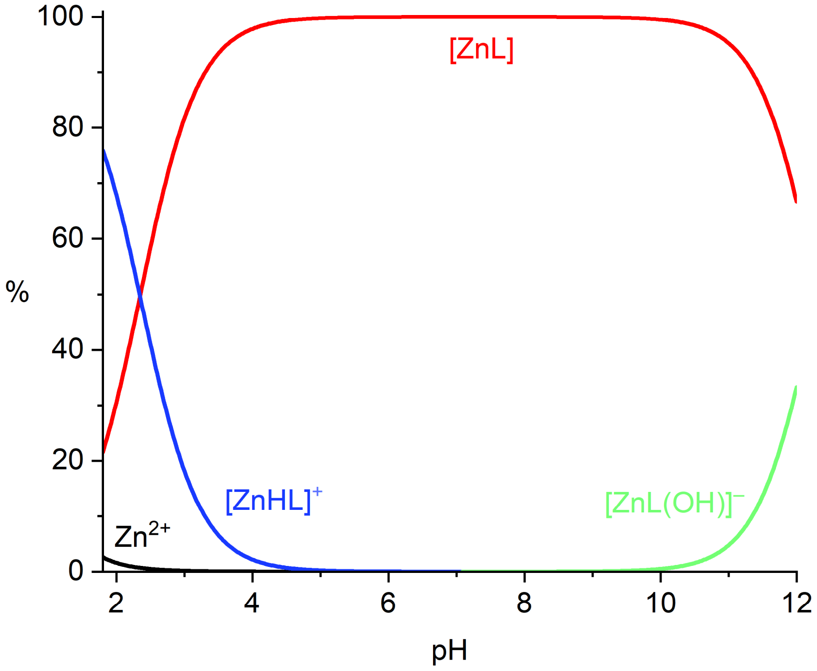

Figure S32. Distribution diagram of the Zn^{II}–**H_2dppa** system ($c_M = c_L = 4$ mM, 25 °C).

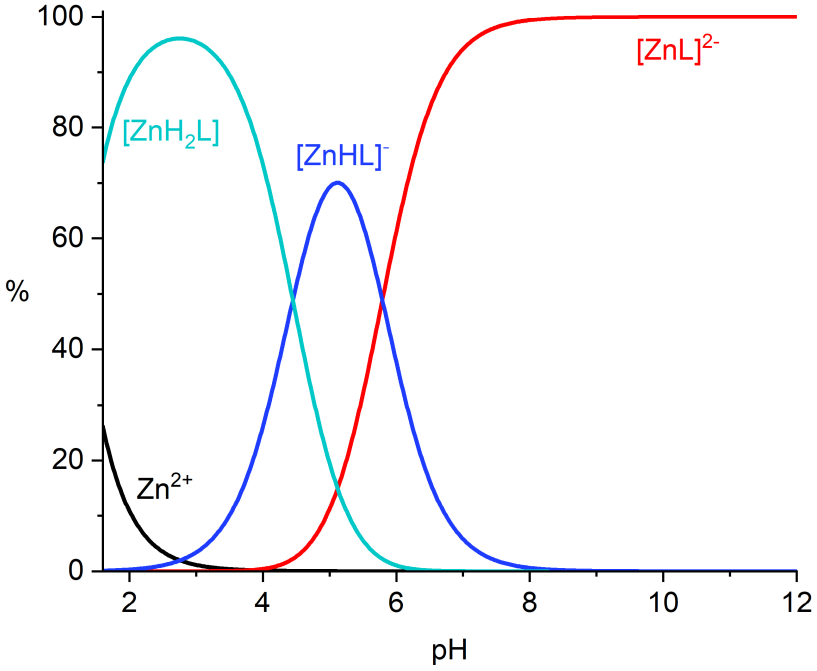

Figure S33. Distribution diagram of the Zn^{II}–**H_4dppp** system ($c_M = c_L = 4$ mM, 25 °C).

Crystallography

Table S3. Crystallographic parameters of the studied compounds (Part 1).

Parameter	(H_4**dppp**)·$3H_2O$	[Ni(**dpph**)Cl_2] ·H_2O·0.5*i*PrOH	[Cu(**dpph**)Cl_2] ·H_2O·0.5*i*PrOH	[Zn(**dpph**)Cl_2] ·H_2O·0.5*i*PrOH	[Ni(**dppa**)(H_2O)$_2$]·$2H_2O$	[Ni(**dppa**)]·$2H_2O$
Formula	$C_{16}H_{28}N_4O_9P_2$	$C_{15.5}H_{22}Cl_2N_4NiO_{1.5}$	$C_{15.5}H_{22}Cl_2CuN_4O_{1.5}$	$C_{15.5}H_{22}Cl_2N_4O_{1.5}Zn$	$C_{18}H_{26}N_4NiO_8$	$C_{18}H_{22}N_4NiO_6$
Colour	colourless	light blue	light blue	colourless	light purple	deep purple
M_r	482.36	417.98	422.81	424.64	485.14	449.10
Crystal system	triclinic	orthorhombic	orthorhombic	orthorhombic	monoclinic	monoclinic
Space group	*P*−1	*Fddd*	*Fddd*	*Fddd*	*C*2/*c*	*C*2/*c*
a (Å)	10.3806(5)	8.5884(5)	8.6379(5)	8.6134(3)	15.8485(5)	29.776(1)
b (Å)	10.5152(5)	28.518(2)	28.721(2)	28.957(1)	14.0168(4)	9.663(3)
c (Å)	10.8179(5)	29.137(2)	28.937(2)	29.024(1)	9.6438(4)	13.606(5)
α (°)	78.849(2)	90	90	90	90	90
β (°)	62.627(1)	90	90	90	108.634(1)	107.757(1)
γ (°)	77.618(2)	90	90	90	90	90
V (Å^3)	1018.08(8)	7136.4(8)	7178.9(7)	7239.1(5)	2030.0(1)	3728.0(2)
Z	2	16	16	16	4	8
D_{calc} (g/m^3)	1.574	1.556	1.565	1.558	1.587	1.600
μ (mm^{-1})	0.274	1.400	1.528	1.665	1.011	1.087
Total refl.	5050	2226	2236	2256	2513	4607
Obsd. refl. [$I > 2\sigma(I)$]	4753	2175	2139	2117	2471	4514
R	0.0278	0.0200	0.0208	0.0219	0.0216	0.0223
R' [$I > 2\sigma(I)$]	0.0297	0.0204	0.0217	0.0239	0.0220	0.0226
wR	0.0719	0.0527	0.0585	0.0552	0.0529	0.0568
wR' [$I > 2\sigma(I)$]	0.0731	0.0531	0.0592	0.0564	0.0532	0.0570
CCDC ref. no.	2516505	2516499	2516502	2516500	2516498	2516504

Table S3. Crystallographic parameters of the studied compounds (Part 2).

Parameter	[Cu(**dppa**)]·2H_2O	{[Cu(H_2**dppp**)]$_2$}·10H_2O·2CH_3COCH_3	[Co(**dppa**)]Cl·4.5H_2O	[Fe(**dppa**)Cl]·4H_2O	{[Ga(**dppa**)]$_4$}Cl_4·17H_2O
Formula	$C_{18}H_{22}CuN_4O_6$	$C_{38}H_{72}Cu_2N_8O_{24}P_4$	$C_{18}H_{27}ClCoN_4O_{8.5}$	$C_{18}H_{26}CFelN_4O_8$	$C_{72}H_{106}Cl_4Ga_4N_{16}O_{33}$
Colour	green-blue	green	orange	yellow	white
M_r	453.93	1275.99	529.81	517.73	2144.40
Crystal system	monoclinic	triclinic	monoclinic	triclinic	triclinic
Space group	*C*2/*c*	*P*−1	*P*2$_1$/c	*P*−1	*P*−1
a (Å)	29.716(1)	11.0889(6)	9.6618(7)	9.059(1)	9.5403(5)
b (Å)	9.7356(4)	11.3906(6)	15.588(1)	9.764(1)	15.2608(8)
c (Å)	13.7443(6)	11.9217(6)	14.686(1)	14.477(2)	15.6879(8)
α (°)	90	94.577(2)	90	72.604(5)	100.388(2)
β (°)	109.119(1)	102.408(2)	105.502(3)	72.489(5)	106.769(2)
γ (°)	90	110.001(2)	90	63.055(5)	91.963(2)
V (Å^3)	3756.9(3)	1362.5(1)	2131.4(3)	1068.4(3)	2142.0(2)
Z	8	1	4	2	1
D_{calc} (g/m^3)	1.605	1.555	1.651	1.609	1.662
μ (mm^{-1})	1.208	0.985	0.988	0.885	1.466
Total refl.	4661	6725	5293	5303	10566
Obsd. refl. [$I > 2\sigma(I)$]	4572	6631	5056	5156	9401
R	0.0271	0.0228	0.0317	0.0373	0.0294
R' [$I > 2\sigma(I)$]	0.0275	0.0231	0.0333	0.0382	0.0343
wR	0.0716	0.0616	0.0802	0.0886	0.0797
w*R'* [$I > 2\sigma(I)$]	0.0719	0.0618	0.0811	0.0891	0.0828
CCDC ref. no.	2516507	2516503	2516508	2516506	2516501

Table S4. Bond lengths (Å) and angles (°) of the metal coordination sphere in the solid state.

	[Ni(**dpph**)Cl_2]<br>·H_2O·0.5*i*PrOH	[Cu(**dpph**)Cl_2]<br>·H_2O·0.5*i*PrOH	[Zn(**dpph**)Cl_2]<br>·H_2O·0.5*i*PrOH
M–N1/N1$^{\#}$ (py)	2.012(1)	2.039(1)	2.121(1)
M–N4/N10	2.182(1)	2.333(1)	2.271(1)
M1–Cl	2.396(1)	2.318(1)	2.388(1)
N1–M–N1$^{\#}$ (py)	85.67(6)	82.75(6)	81.75(6)
N4–M–N4$^{\#}$	152.13(6)	147.65(6)	144.61(6)
Cl–M–Cl$^{\#}$	90.93(2)	93.33(2)	92.83(2)
	[Ni(**dppa**)(H_2O)$_2$]·2H_2O	[Ni(**dppa**)]·2H_2O	[Cu(**dppa**)]·2H_2O
M–N1/N1$^{\#}$/N7 (py)	2.004(1)	1.993(1)/1.999(1)	1.982(1)/2.151(1)
M–N4/N4$^{\#}$/N10	2.222(1)	2.148(1)/2.128(1)	2.364(1)/2.169(1)
M1–X [a]	2.014(1)	2.066(1)/2.043(1)	1.943(1)/2.017(1)
N1–M–N1$^{\#}$/N7 (py)	84.58(5)	86.74(4)	83.25(5)
N4–M–N4$^{\#}$/N10	153.88(5)	155.53(4)	148.40(4)
X–M–X [a]	94.17(6)	90.55(3)	92.33(4)
	{[Cu(H_2**dppp**)]$_2$}<br>·10H_2O·2CH_3COCH_3	[Cu(H_2**dppa**)Cl_2] [c,d]	[Cu_4(H**dppa**)$_4$(ClO_4)$_4$] [c]
M–N1/N7 (py)	2.005(1)/2.053(1)	1.98–2.07	1.905/2.191
M–N4/N10	2.340(1)/2.339(1)	2.23–2.40	2.253/2.313
M1–X [a]	2.031(1)/1.932(1)	2.29–2.38	2.191/1.905
N1–M–N1$^{\#}$/N7 (py)	81.90(4)	81–83	82.01
N4–M–N4$^{\#}$/N10	149.85(4)	147–148	147.26
X–M–X [a]	93.43(4)	94–95	85.69
	[Co(**dppa**)]Cl·4.5H_2O	[Fe(**dppa**)Cl]·4H_2O	{[Ga(**dppa**)]$_4$}Cl_4·17H_2O [d]
M–N1/N7 (py)	1.838(1)/1.838(1)	2.088(2)/2.258(2)	2.037(2)/2.108(2)<br>2.033(2)/2.128(2)
M–N4/N10	1.945(1)/1.948(1)	2.315(2)/2.311(2)	2.244(2)/2.311(1)<br>2.323(2)/2.235(2)
M1–O [b]	1.896(1)/1.920(1)	2.079(1)/2.093(1)	2.030(1)/2.121(1)<br>1.954(1)/2.400(1)
N1–M–N1/N7 (py)	93.51(6)	77.35(6)	84.81(6)<br>82.02(6)
N4–M–N4/N10	167.95(6)	138.85(6)	143.09(6)<br>145.42(5)
O–M–O [b]	89.62(5)	72.79(5)	73.13(5)<br>89.26(5)
Fe–Cl [e] / Ga–O [f]	–	2.286(1) [e]	1.972(1) [f]<br>1.927(1) [f]

[a] X = Cl or H_2O or pendant arm oxygen atom; [b] O = pendant arm oxygen atom; [c] Ref. 4; [d] Two independent complex molecules; [e] Coordinated chloride ion; [f] Pendant oxygen atom of the neighbouring complex molecule.

References

[1] A. Jouaiti and M. W. Hosseini, *Helv. Chim. Acta*, 2009, **92**, 2497.

[2] Y.-J. Liu, Y.-Z. Han, Y.-Z. Zhang, W. Zhang, W.-Z. Lai and Y. Wang, *Chem. Commun.*, 2017, **53**, 3189.

[3] A. J. Wessel, J. W. Schultz, F. Tang, H. Duan and L. M. Mirica, *Org. Biomol. Chem.*, 2017, **15**, 9923.

[4] A. K. Sharma, J. W. Schultz, J. T. Prior, N. P. Rath and L. M. Mirica, *Inorg. Chem.,* 2017, **56**, 13801.
